# PINK1-PTEN axis promotes metastasis and chemoresistance in ovarian cancer via non-canonical pathway

**DOI:** 10.1186/s13046-023-02823-w

**Published:** 2023-11-09

**Authors:** Fang Zheng, Jiamin Zhong, Kelie Chen, Yu Shi, Fang Wang, Shengchao Wang, Song Tang, Xiaoyu Yuan, Zhangjin Shen, Sangsang Tang, Dajing Xia, Yihua Wu, Weiguo Lu

**Affiliations:** 1 Women’s Reproductive Health Laboratory of Zhejiang Province, Women’s Hospital, Zhejiang University School of Medicine, Hangzhou, China; 2 Department of Toxicology of School of Public Health and Department of Gynecologic Oncology of Women’s Hospital, Zhejiang University School of Medicine, Hangzhou, China; 3 State Key Laboratory for Diagnosis and Treatment of Infectious Diseases, National Clinical Research Center for Infectious Diseases, Collaborative Innovation Center for Diagnosis and Treatment of Infectious Diseases, the First Affiliated Hospital, School of Medicine, Zhejiang University, Hangzhou, China; 4 Department of Gynecologic Oncology of Women’s Hospital, Zhejiang University School of Medicine, Hangzhou, China; 5 Cancer Center, Zhejiang University, Hangzhou, China; 6 Research Unit of Intelligence Classification of Tumor Pathology and Precision Therapy, Chinese Academy of Medical Sciences (2019RU042), Hangzhou, China; 7 Zhejiang Provincial Clinical Research Center for Obstetrics and Gynecology, Hangzhou, China

**Keywords:** Ovarian cancer, Chemoresistance, Metastasis, Phosphorylation, PINK1, PTEN

## Abstract

**Background:**

Ovarian cancer is commonly associated with a poor prognosis due to metastasis and chemoresistance. *PINK1 (PTEN-induced kinase 1)* is a serine/threonine kinase that plays a crucial part in regulating various physiological and pathophysiological processes in cancer cells.

**Methods:**

The ATdb database and "CuratedOvarianData" were used to evaluate the effect of kinases on ovarian cancer survival. The gene expression in ovarian cancer cells was detected by Western blot and quantitative real-time PCR. The effects of gene knockdown or overexpression in vitro were evaluated by wound healing assay, cell transwell assay, immunofluorescence staining, immunohistochemistry, and flow cytometry analysis. Mass spectrometry analysis, protein structure analysis, co-immunoprecipitation assay, nuclear-cytoplasmic separation, and in vitro kinase assay were applied to demonstrate the PINK1-PTEN (phosphatase and tensin homolog) interaction and the effect of this interaction. The metastasis experiments for ovarian cancer xenografts were performed in female BALB/c nude mice.

**Results:**

PINK1 was strongly associated with a poor prognosis in ovarian cancer patients and promoted metastasis and chemoresistance in ovarian cancer cells. Although the canonical PINK1/PRKN (parkin RBR E3 ubiquitin protein ligase) pathway showed weak effects in ovarian cancer, PINK1 was identified to interact with PTEN and phosphorylate it at Serine179. Remarkably, the phosphorylation of PTEN resulted in the inactivation of the phosphatase activity, leading to an increase in AKT (AKT serine/threonine kinase) activity. Moreover, PINK1-mediated phosphorylation of PTEN impaired the nuclear import of PTEN, thereby enhancing the cancer cells’ ability to resist chemotherapy and metastasize.

**Conclusions:**

PINK1 interacts with and phosphorylates PTEN at Serine179, resulting in the activation of AKT and the inhibition of PTEN nuclear import. PINK1 promotes ovarian cancer metastasis and chemotherapy resistance through the regulation of PTEN. These findings offer new potential therapeutic targets for ovarian cancer management.

**Supplementary Information:**

The online version contains supplementary material available at 10.1186/s13046-023-02823-w.

## Background

Ovarian cancer continues to be the second most common malignancy among women aged above 40 years and the most lethal gynecologic cancers globally [1]. Despite significant progress in targeted molecular therapies, the primary treatment for advanced ovarian cancer is a combination of surgery and chemotherapy [2]. Nonetheless, most patients will relapse with chemoresistance, portending a very poor prognosis [3]. In addition, metastasis is an important cause of higher mortality associated with ovarian cancer [4].

Autophagy is a critical cellular process that has been linked to multiple diseases, particularly to cancer [5, 6]. In recent years, there has been substantial focus on exploring the molecular mechanisms of autophagy, and kinases have surfaced as crucial regulators of the autophagy initiation and regulation, as well as cancer. *ULK1/2 (unc-51 like autophagy activating kinase 1/2)*, for instance, phosphorylates *Beclin1*, thereby participating in the initiation of autophagy [7] and regulating tumors.

Discovered in 2001*, PTEN-induced putative kinase 1* (*PINK1*) is a serine/threonine kinase whose upregulation was first observed as a consequence of PTEN activity in cancer cells [8, 9]. Although PINK1 is well-studied in Parkinson’s disease [10], research has also uncovered its significance in cancer cell biology. Initially identified as a key participant in the mitochondrial quality control system via mitophagy, an autophagic process, further evidence suggests that PINK1 is also involved in various subcellular pools to regulate cancer cell survival and chemoresistance [11, 12]. A recent meta-analysis conducted on individuals with ovarian cancer found a strong correlation between elevated PINK1 mRNA levels and unfavorable prognosis [13]. While most previous studies have focused on PINK1’s function of phosphorylating Parkin in mitophagy [14], an increasing number of studies show that PINK1 is also capable of regulating tumors independent of Parkin [15]. Several substrates, such as *tp53 (tumor protein p53)*, *MFN2* (*mitofusin 2)*, *Ubiquitin*, were found to be phosphorylated by PINK1 to modulate cancers [16–19].

Identified in 1997, PTEN is a potent tumor suppressor [20, 21]. It involves in various biological processes of cancer, such as cell survival, migration, proliferation, and metabolism [22]. Recent discoveries indicate that PTEN/PI3K (phosphatidylinositide 3-kinases)/AKT signaling pathway plays a role in modulating multidrug resistance of cancers [23]. PTEN can be modified by acetylation, oxidation, phosphorylation, and ubiquitination. Several kinases have been identified as playing a role in PTEN phosphorylation, including *casein kinase 2 (CK2)*, *glycogen synthase kinase-3 beta (GSK3β)*, and *ras homolog family member A (RhoA) kinase* [24–29]. In general, the phosphorylation of PTEN enhances its stability but simultaneously diminishes its activity [28–30].

Considering that PINK1 has been identified as a target of PTEN, we investigated whether PINK1 could regulate PTEN to form a regulatory loop. In this study, we utilized a series of assays and identified the PINK1-PTEN interaction. Additionally, the phosphatidylinositol (PtdIns) (4,5)P2-binding domain (PBD) domain of PTEN is responsible for binding to PINK1. We also found that PINK1 phosphorylates PTEN at Ser179, thereby regulating its nuclear localization and activating AKT in ovarian cancer. Our study suggests that PINK1 contributes to ovarian cancer metastasis and chemotherapy resistance through the regulation of PTEN via a non-canonical pathway. Our study presents a promising idea for predicting and intervening in the progression of chemoresistance and metastasis in ovarian cancer.

## Materials and methods

### Public datasets retrieval

The ATdb database [31] and "CuratedOvarianData" [32] were applied to screen and evaluate the effect of autophagy associated kinases on ovarian cancer survival. The Kaplan–Meier Plotter database [33] (http://kmplot.com) was used to evaluate the association between PINK1 expression level and overall survival (OS) in patients with ovarian cancer. Ovarian cancer samples from each dataset were classified into two categories (high versus low PINK1 expression) with the auto select best cutoff. The hazard ratio (HR) and 95% confidence interval (CI) were calculated by the log-rank test in Kaplan–Meier plotter. A random-effect model was employed to pool the HR comparing the high PINK1 expression level with the low categories and draw forest plots in meta-analysis. The datasets used in Kaplan–Meier Plotter are shown in the Supplementary material for methods. The Human Protein Atlas (proteinatlas.org) is an open-access protein database [34] and the expression data of PINK1 in ovarian cell lines and the expression of PRKN in diverse tissues were downloaded from this database.

### Patients and specimens

Ovarian cancer tissues from clinical patients were obtained from Women’s Hospital School of Medicine, Zhejiang University (Hangzhou, China) with Institutional Review Board (IRB) approval (Approval Number IRB-20200135-R). We conducted immunohistochemistry (IHC) to compare the expression levels of PINK1 in primary and metastatic ovarian lesions. Informed consent from all enrolled patients was obtained. The procedures carried out in this study were in accordance with the Declaration of Helsinki.

### Quantitative real-time PCR, Co- Immunoprecipitation (Co-IP) assay, and Western blotting

Trizol reagent (B511311, Sangon Biotech, China) was applied to extract total RNA from cells. Protein A/G PLUS-Agarose (sc-2003, Santa Cruz) was used in the co-IP assay. The details of Western blotting, qRT-PCR and co-IP assay are shown in the Supplementary material for methods. Phos-tag™ Acrylamide (304–93521, NARD, Japan) was applied in phos-tag SDS–PAGE gels. Antibody information is listed in Table S1.

### Animal study

The metastasis experiments for ovarian cancer xenografts were performed in 5-week-old female BALB/c nude mice. The mice were randomly separated into 4 groups (each group *n* = 6). Cisplatin (DDP, 232120, sigma, USA) was dissolved in a 0.9% saline solution. Stable cells included shPINK1 and negative control SKOV3 cells were resuspended in PBS (phosphate buffered saline). 1.5 × 10^6^ SKOV3 cells in 150 mL PBS for each mouse were intraperitoneally injected into the right flank. DDP (4mg/kg) or saline was intraperitoneally injected into the nude mice every 3 days after two weeks. The mice were euthanized six weeks later, and tumor tissues were obtained for further analysis. The study was approved by the Ethics Committee of Zhejiang University (Approval Number ZJU20220292).

### Wound healing assay, cell transwell assay, and cell proliferation assay

Wound scratch assays and cell transwell assays were conducted as described in our previous study [35]. Digital microscope (Carl Zeiss Jena, Germany) was applied to capture image. The proliferation ability was assessed using the EdU (5-ethynyl-2'-deoxyuridine) Cell Proliferation Kit (C0078, Beyotime, China) and Cell counting Kit-8 (CCK-8) assay (C0037, Beyotime, China). See more details in supplementary materials.

### Cell survival assay and IC_50_ determination

Cisplatin (Sigma, USA) was diluted with a 0.9% saline solution to a series of expected concentrations. SKOV3 or A2780 cells were seeded at a density of 5 000 cells in 96-well plates. After adhesion, they were treated with different concentrations of cisplatin. After 48 h treatment with cisplatin, the cell viability was evaluated using the CCK-8. Optical density OD_450_ value was recorded using a Microplate Reader (SynergyMx M5, USA). The half maximal inhibitory concentration (IC_50_) was determined as the concentration causing a 50% decrease in growth compared to cells treated with solvent. Each Group included three replicate wells.

### Mass spectrometry analysis and protein structure analysis

Immunoprecipitates were separated by SDS-PAGE and analyzed by liquid chromatograpgy-tandem mass spectrometry (Beijing Qinglian Biotech Co.,Ltd, China). The Z-docking server was used for simulating molecular docking. For more details, please see the Supplementary material for methods.

### Construction and transfection of plasmid, siRNA, and short hairpin RNA

The small interfering RNA (siRNA) was transfected using the Powerfect reagent. The full-length and PTEN-truncated plasmids were transfected using Lipofectamine 3000 (Thermo Fisher Scientific, USA). Short hairpin RNA (shRNA) was used to construct stable PINK1 knockdown in SKOV3 cells. The sequences of siRNAs and shRNAs are listed in the Table S2. HEK293T cells were used to transfect plasmid and packaging vectors using Lipo3000. And the medium supernatant containing the virus was added to infect SKOV3 cells, along with 7μg/ml Polybrene (C0351, Beyotime, China). Then these cells were selected after 48 h by puromycin. Detailed procedures are shown in Supplementary materials for methods.

### Immunofluorescence (IF) staining, and Immunohistochemistry (IHC) staining

IHC was performed using the SP Rabbit & Mouse HRP Kit (DAB) (CW2069S, CWBIO, China). The image acquisition was applied with a fluorescence microscopy (bx63, Olympus, Japan) or Inverted laser scanning Confocal microscope (FV1000, Olympus, Japan). The immunoreactivity was scored based on the percentage of positively stained ovarian cells and the intensity of staining evaluated in five random fields. The percentage of positively stained ovarian cancer cells in sections was graded as follows: 0, no positive cells; 1, ≤ 25%; 2, 26–50%; 3, 51–75%; 4, ≥ 76%. The staining intensity was recorded as follow: 0, no staining; 1, light brown; 2, brown; 3, dark brown. The staining index (SI) was calculated as follows: SI = staining intensity × percentage of positively stained cells [36, 37]. In addition, cut-off value of PINK1 expression was chosen by which best discriminated overall survival to categorize the high and low staining group [38, 39]. Refer to Supplementary materials for further details on the methods.

### In vitro kinase assay, cytoplasmic and nuclear protein extraction, and flow cytometry analysis

The reaction system for in vitro kinase assay consisted of purified PINK1 and PTEN, which were mixed with kinase buffer (#9802, Cell Signaling Technology). Nuclear and Cytoplasmic Protein Extraction Kit (P0027, Beyotime) was applied for cytoplasmic and nuclear protein extraction. Annexin V-FITC/PI apoptosis kit (AP101-100, MultiSciences, China) was used to detect the apoptotic cells. See more details in Supplementary materials for methods.

### Statistical analyses

Statistical analyses were applied using the GraphPad Prism 8 software and R software (Version 4.1.0, The R Foundation for Statistical Computing, Vienna, Austria). For consistency, all results were presented as mean ± standard error of mean (SEM) unless stated otherwise, and they were derived from at least three independent experiments. A two-tailed student's t-test was conducted for determine the statistical significance. The Kaplan–Meier method was applied in survival analysis, and the R package survminer was used to draw the plot. Volcanic plot was generated with R package pheatmap(Version 1.0.12). Heterogeneity between individual studies was evaluated by χ^2^ test and I^2^ test, and *p* ≤ 0.05 and/or I^2^ > 50% indicates the heterogeneity is significant. A random-effect model was used when the heterogeneity was significant and a fixed-effect model was applied with non-significant heterogeneity. A *p*-value < 0.05 was considered statistically significant in all analyses.

## Results

### High expression of PINK1 is associated with poor prognosis of ovarian cancer

**Fig. 1:**
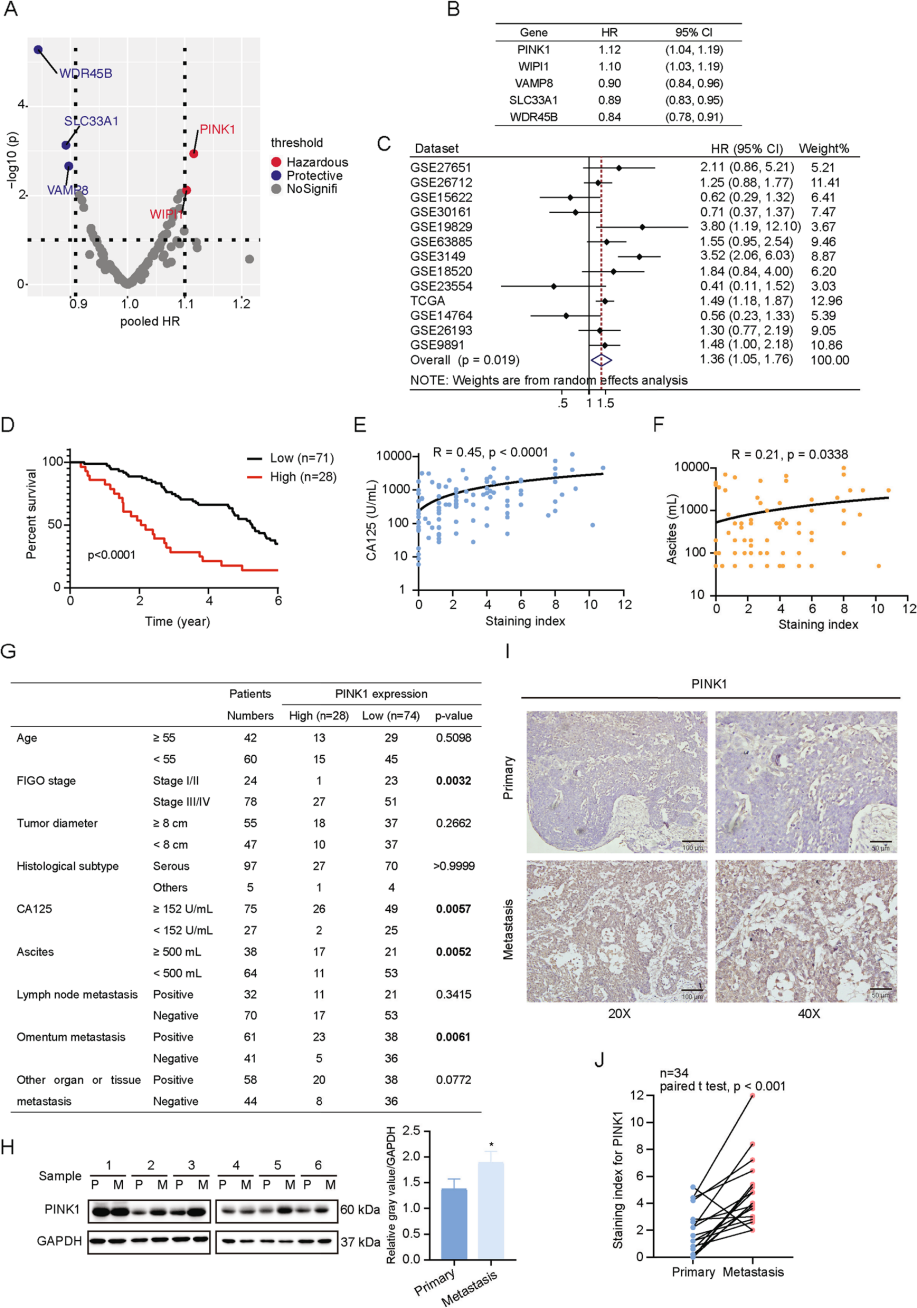
PINK1 is associated with poor prognosis of ovarian cancer. **A** The correlation between autophagy-related kinase and ovarian cancer patients’ prognosis. **B** The Hazard Ratio (HR) and 95% confidence interval (CI) of the significant correlation between autophagy-related kinase with ovarian cancer patients’ prognosis. **C** Forest plot of Hazard Ratios for evaluating the association between PINK1 expression and overall survival of ovarian cancer patients. The data included was obtained from Kaplan–Meier website and was stratified based on PINK1 expression. The red dotted line represents the pooled HR of meta-analysis. **D** A Kaplan–Meier plot was used to display overall survival curves of ovarian cancer patients from a local cohort stratified based on PINK1 expression levels. **E** The correlation between PINK1 expression and CA125 serum level. **F** The correlation between PINK1 expression and ascites volume. **G** Association between PINK1 expression and clinicopathological features. **H** Western blot analysis for PINK1 protein levels in six paired primary and metastatic ovarian cancer tissues. **I** Representative images of immunohistochemistry (IHC) staining depicting the expression of PINK1 in human samples of ovarian carcinoma in primary and metastatic tissue. **J** Score of IHC staining against PINK1 in paired primary and metastatic ovarian cancer specimens. * *p* < 0.05. Values are mean ± SEM. Data are representative of 3 independent experiments

The close correlation between autophagy and tumors prompted us to apply the ATdb database [31] established by our team (http://www.bigzju.com/ATdb/#/) and an R package "CuratedOvarianData" [32] to screen the autophagy-related kinases that correlated with the prognosis of ovarian cancer patients. Our analysis revealed that a high expression of PINK1 was significantly associated with a worse prognosis in patients with ovarian cancer (Fig. 1A, B, Fig. S1A). To further validate the effect of PINK1 expression on prognosis of ovarian cancer, multiple datasets were obtained from Kaplan–Meier Plotter website (Fig. 1C). A comprehensive description of the patient cohort was shown in Table S3. Meta-analysis was conducted to depict the results of the relativity between PINK1 expression and ovarian cancer prognosis. The forest plot suggested that high PINK1 expression rather than WIPI1 was strongly correlated with poor outcome (Fig. 1C, Fig. S1B). Consistent with our local cohort data (Fig. 1D), these results point to PINK1 overexpression as an unfavorable indicator for ovarian cancer. Moreover, a positive relationship was observed between PINK1 levels and serum expression of CA125 (Fig. 1E) and ascites volume (Fig. 1F), but not tumor diameter (Fig. S1C). We also found that PINK1 expression may associated with FIGO stage and omentum invasion (Fig. 1G). Collectively, these observations demonstrate that PINK1 is closely associated with poor prognosis in ovarian tumor.

Given that poor prognosis of ovarian cancer is often associated with metastasis, and high PINK1 expression was found to be correlated with greater omentum metastasis (Fig. 1G), we went ahead to assess PINK1 protein levels within primary and metastatic ovarian cancer tissues. Immunoblot analysis (Fig. 1H) demonstrated that PINK1 protein levels were significantly higher in metastatic samples. Additionally, IHC analysis also revealed up-regulation of PINK1 level in metastatic tissues (Fig. 1I, J).

The collective findings reveal that PINK1 plays a significant role in the metastasis and unfavorable prognosis of ovarian cancer.

### PINK1 promotes metastasis of ovarian cancer in vitro

To explore the role of PINK1 in the development of ovarian tumor, we checked the expression levels of PINK1 in several cell lines including A2780, ES-2, SKOV3, OVCAR3 cancer cells, and ovarian epithelial cell line IOSE80 (Fig. S2A-D). Among these cell lines, the protein expression levels of PINK1 were lowest in A2780 cells and highest in SKOV3 cells. Therefore, these two particular cell lines were selected to determine the impact of PINK1 on ovarian cancer. Specifically, A2780 and SKOV3 cells were transfected with Flag-tagged PINK1 over-expression plasmid and PINK1-targeting siRNA respectively to observe their effects on the cells. The efficiency of the transfection was demonstrated (Fig. S2E, F).

**Fig. 2:**
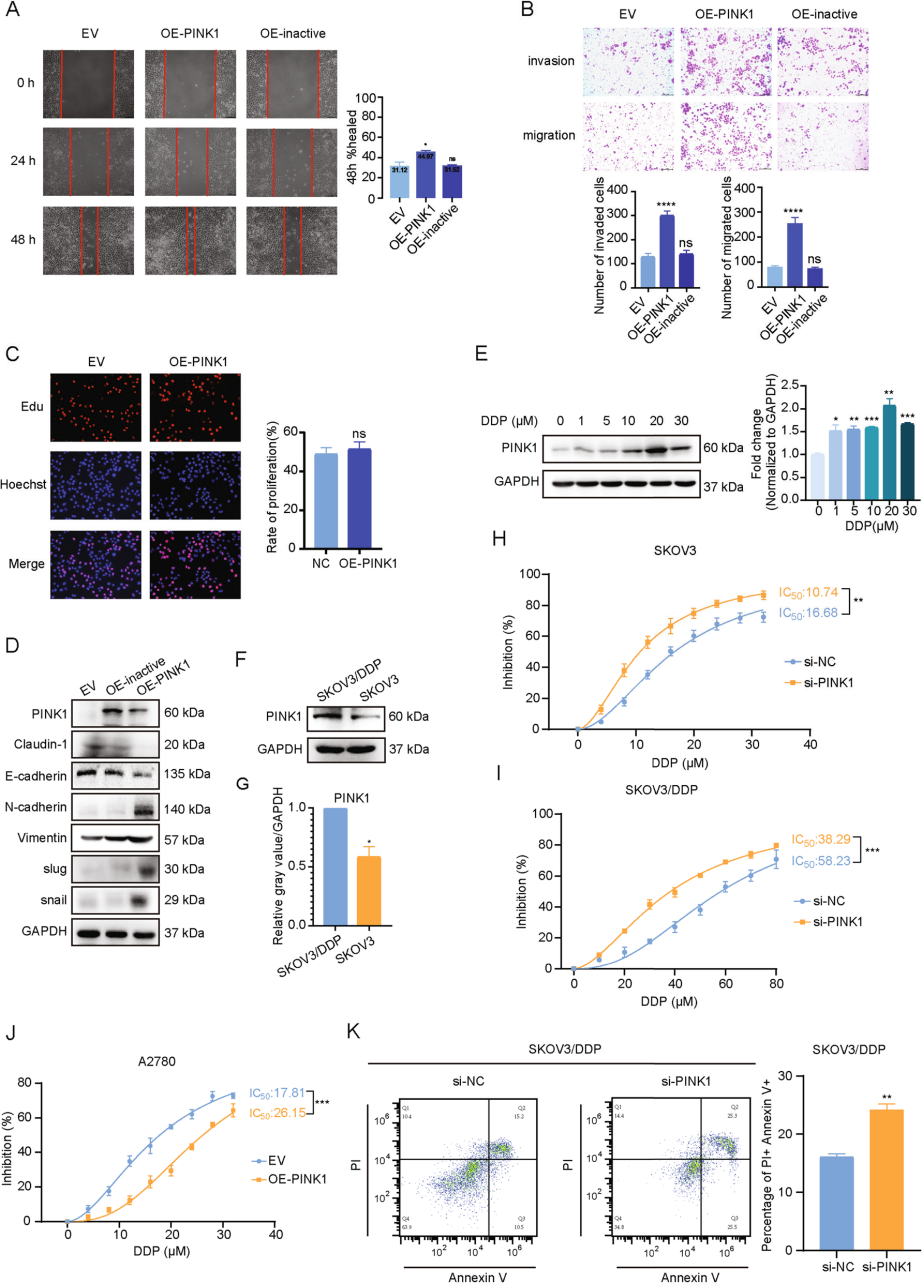
PINK1 promotes metastasis and chemoresistance of ovarian cancer in vitro. **A** Cells were transfected with over-expression plasmid for 24 h. Representative images of the wound scratch assay utilizing the A2780 cell lines after scratching 24 and 48 h. The histograms on the right show the quantitative results of the healing percentage after 48 h of three independent replicates. **B** Effects of PINK1 on A2780 cell invasion (upper) and migration (bottom). Cells were transfected by PINK1 over-expression plasmid for 24 h and then measured using transwell assay with (invasion) or without (migration) Matrigel after incubation for 24 h. Invasion or migration cells were fixed, stained, photographed, and counted in 6 random views. The quantitative analysis of invade or migrated cells were shown on the bottom. **C** Representative images (left) and quantitative analysis (right) of proliferating A2780, which were transfected by Flag-PINK1 over-expression vector for 48 h and then assessed by EdU kit assay. **D** Western blot analysis of EMT markers. A2780 cells were transfected by PINK1 over-expression plasmid. **E** The protein (left) and mRNA (right) levels of PINK1 in ovarian cancer cells treated with DDP as indicated concentrations for 24 h. **F** and **G** The protein levels of PINK1 in SKOV3 cell and SKOV3/DDP cell by western blot analysis (**F**) and the quantification analysis (**G**). **H** and **I** IC_50_ values of cisplatin are tested in PINK1 targeting siRNA-transfected SKOV3 cells (**H**) and SKOV3/DDP cells (**I**) by CCK-8 assay. **J** IC_50_ values of cisplatin are tested in PINK1 over-expressed A2780 cells. Results were shown as mean ± standard deviation (SD) in **H-J**. **K** FCM analysis on SKOV3/DDP cells treated with PINK1 targeting siRNA and cisplatin (20μM) for 48 h. ns, no statistical significance. * *p* < 0.05, ** *p* < 0.01, *** *p* < 0.001, **** *p* < 0.0001. Values are mean ± SEM. Data are representative of 3 independent experiments

To evaluate the impact of PINK1 on the migration and invasion ability of ovarian cancer cells in vitro, scratch experiments and matrigel invasion assays were performed. Our results demonstrated that over-expression of PINK1 significantly enhanced the migration and invasion ability in A2780 cells (Fig. 2A, B). In contrast, PINK1 knockdown reduced the migration and invasion ability of SKOV3 cells (Fig. S2G, H). Then, we evaluated the effect of PINK1 on the proliferative ability of ovarian cancer cells using EdU (Fig. 2C, Fig. S3A) and CCK-8 assays (Fig. S3B, C). Our findings indicated that PINK1 had no statistically significant effect on ovarian cancer cell proliferation.

Given the important role of epithelial-mesenchymal transition (EMT) in tumor metastasis, we investigated whether PINK1 could promote EMT in ovarian cancer cells. Our analysis revealed that PINK1 over-expression significantly down-regulated epithelial markers such as E-cadherin. In contrast, the mesenchymal biomarkers including N-cadherin, and the key EMT transcription factors slug and snail, were increased (Fig. 2D). As anticipated, knock down of PINK1 led to the opposite result (Fig. S3D).

We next evaluated whether the kinase activity of PINK1 is necessary for its effect on ovarian cancer metastasis by over-expressing a PINK1 kinase inactive mutant plasmid [40] in A2780 cells. Compared with over-expression of PINK1 wild type (WT), kinase inactive mutant failed to enhance the cell migration and invasion ability (Fig. 2A, B). Additionally, we demonstrated that over-expression of PINK1 WT led to the promotion of EMT, whereas the kinase inactive mutant did not (Fig. 2D). These results indicate that the pro-metastasis ability of PINK1 predominantly depends on its kinase activity.

Collectively, these results provide strong evidence supporting the notion that PINK1 promotes ovarian cancer metastasis through its kinase activity.

### PINK1 knockdown enhances cisplatin sensitivity

Interestingly, we observed a significant reduction in overall survival and progression-free survival among patients with high PINK1 expression in advanced stages (stage3-4) based on data from Kaplan–Meier Plotter database (Fig. S3E, F). These results further support the notion that PINK1 is highly associated with malignancy and poor prognosis in ovarian tumors. As drug resistance is often the primary cause of final relapse and poor prognosis in advanced-stage patients [41], we conducted further investigations to determine whether PINK1 contributes to chemotherapy resistance. Considering cisplatin is one of the most effective chemotherapy drugs for ovarian cancer patients, cisplatin was selected for further exploration.

We found that mRNA and protein levels of PINK1 were up-regulated upon cisplatin exposure in a concentration-dependent manner (Fig. 2E). To further investigate the role of PINK1 in cisplatin resistance, cisplatin-resistant cancer cells (SKOV3/DDP) were introduced. Western blot analysis demonstrated a significant increase in PINK1 protein expression in SKOV3/DDP cells (Fig. 2F, G), which suggests the association of PINK1 with cisplatin resistance in ovarian cancer. To confirm these findings, CCK-8 assay was conducted to evaluate the inhibitory effect of cisplatin in cells within PINK1 knockdown or overexpression. In SKOV3 and SKOV3/DDP cells, the IC_50_ of cisplatin was significantly decreased after PINK1 knockdown(SKOV3 16.68 μM vs. 10.74μM; SKOV3/DDP 58.23 μM vs. 38.29μM) (Fig. 2H, I). Similar results were observed in A2780 cells, where overexpression of PINK1 significantly increased the IC_50_ (17.81μM vs. 26.15μM) (Fig. 2J). Moreover, flow cytometry analysis revealed an increase in apoptosis level upon cisplatin treatment following PINK1 knockdown (Fig. 2K).

These results collectively indicate the involvement of PINK1 in ovarian cancer resistance to cisplatin.

### PINK1 interacts with PTEN

**Fig. 3:**
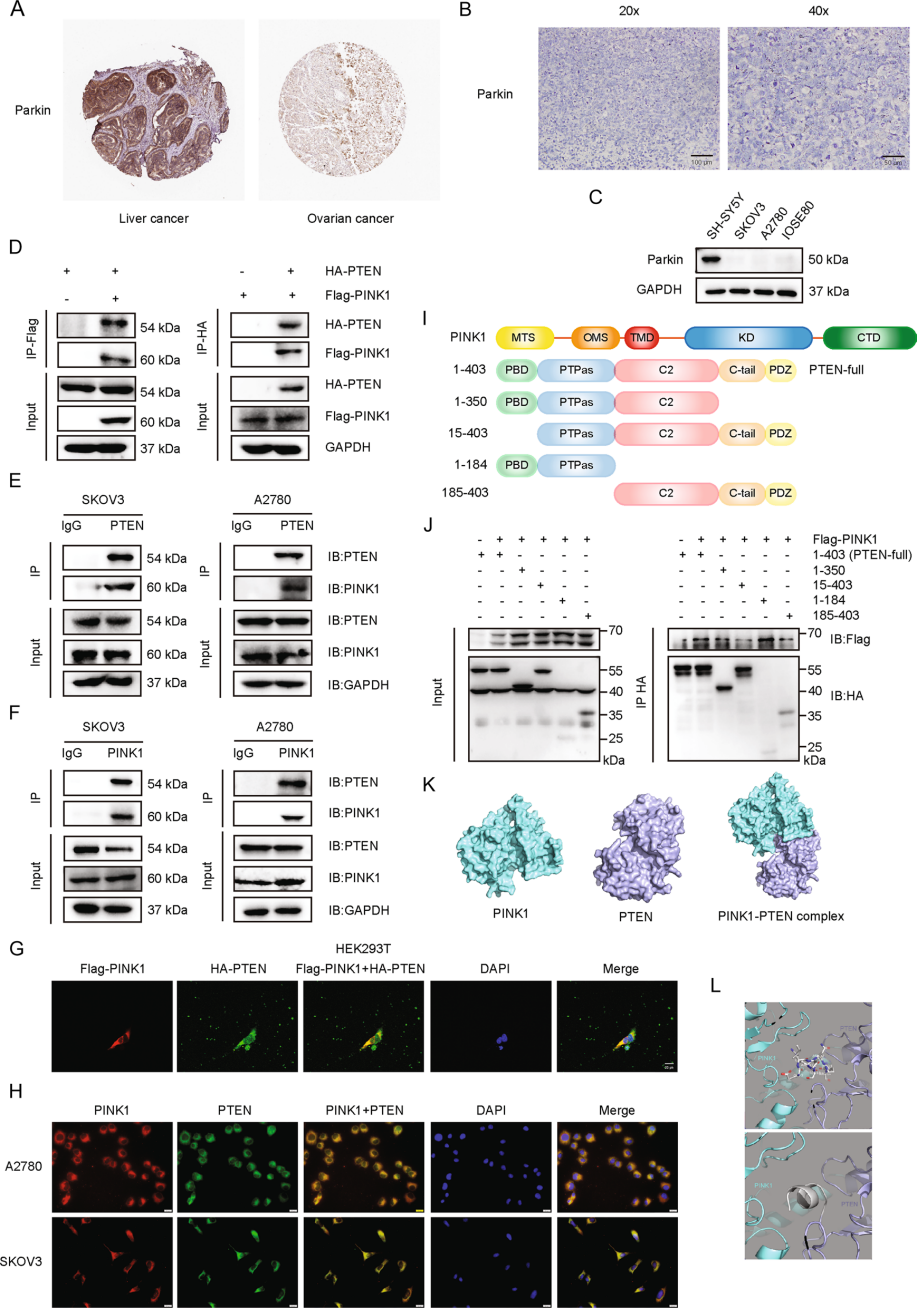
PINK1 interacts with PTEN. **A** Representative IHC image of Parkin from ovarian cancer patient and liver cancer patient downloaded from The Human Protein Atlas. **B** Representative IHC image of Parkin from local ovarian cancer patient. **C** Western blot for Parkin expression in four cell lines. **D** Immunoblotting analysis of Flag-PINK1 and HA-PTEN expression in a co-IP assay performed using Protein A/G PLUS-Agarose and anti-Flag (left) or anti-HA (right) primary antibody in HEK293T cells. **E** and **F** Immunoblotting analysis of endogenous PINK1 and PTEN expression in a co-IP assay performed in A2780 and SKOV3 cells with Protein A/G PLUS-Agarose and anti-PTEN (**E**) or anti-PINK1 (**F**) primary antibody. **G** Confocal microscopy detection of the co-localization of Flag-PINK1 (red) and HA-PTEN (green) in HEK293T cells. Nuclei were stained using DAPI (blue). DAPI, 4’,6-diamidino-2-phenylindole. **H** Immunofluorescence assay of the co-localization of endogenous PINK1 (red) and PTEN (green) in A2780 and SKOV3 cells. **I** Schematic diagram of PINK1 and truncated structure of PTEN. PBD, phosphatidylinositol (PtdIns) (4,5)P2-binding domain; PTPas, phosphatase domain; C2, C2 lipid/membrane-binding domain; C-tail, containing Pro, Glu, Ser and Thr (PEST) sequences; PDZ, PDZ-binding motif. MTS, mitochondrial targeting sequence; OMS, outer membrane localization signal; TMD, transmembrane domain; KD, kinase domain; CTD, C-terminal domain. **J** Flag-PINK1 was expressed with HA-PTEN (full length) or PTEN deletion mutants in HEK293T cells. Total cell lysates were subjected to HA affinity purification and analyzed by immunoblotting with anit-Flag and anti-HA antibodies. **K** Molecular docking model of PINK1/PTEN interaction by Z-DOCK. **L** Diagram of binding mode of PINK1 (green) and PTEN (purple) predicted by Z-docking server. The predicted binding sites are shown in stick mode in the upper panel and in cartoon mode in the bottom panel (grey). PTEN residues 1–14 are located at the binding interface of two proteins and may form contact with PINK1. Data are representative of 3 independent experiments

Our study has shown that PINK1 promoted tumor metastasis and drug resistance dependent on its kinase activity. We next investigated whether the canonical PINK1/Parkin pathway played a role in ovarian cancer development and chemoresistance, but low levels of Parkin in ovarian cancer were observed via sample data retrieved from The Human Protein Atlas (Fig. S4A). In addition, the IHC analysis showed that the expression level of Parkin was extremely low or even not detected in ovarian cancer compared to liver cancer (Fig. 3A, B). Parkin expression in ovarian cancer cell lines was minimal compared to SH-SY5Y cells (Fig. 3C). Therefore, we sought to identify novel substrates of PINK1. Given previous findings that the PINK1 gene is initially identified as an up-regulated target of PTEN in cancer cells, we hypothesized that PINK1 could interact with and regulate PTEN to form a loop. To test our hypothesis, we immunoprecipitated proteins with an anti-PINK1 antibody in SKOV3 cells and identified PTEN using mass spectrometry analysis (Fig. S4B). Additionally, prediction analysis [42–44] of molecular interaction also supported the interaction between the two proteins (Fig. S4C-E).

To verify the physical interaction between PINK1 and PTEN, we overexpressed Flag-PINK1 and HA-PTEN in HEK293T cells and performed a co-immunoprecipitation (co-IP) analysis. Using Protein A/G PLUS Agarose and IP with anti-Flag primary antibody, we detected PTEN, and vice versa, indicating their association with each other (Fig. 3D). We further validated the interaction between endogenous PINK1 and PTEN in ovarian cancer cells using co-IP with an anti-PINK1 or anti-PTEN antibody in A2780 and SKOV3 cell lines. As shown in Fig. 3E, PINK1 was pulled down by PTEN with anti-PTEN primary antibody. Similarly, PTEN was pulled down by PINK1 (Fig. 3F). To explore whether the interaction of PINK1 and PTEN is universal in tumor cells, we repeated co-IP analysis in the colorectal cancer cell line SW480 and confirmed the extensive existence of the interactions between PINK1 and PTEN (Fig. S4F, G). To visualize the PINK1/PTEN interaction, Flag-PINK1 and HA-PTEN were over-expressed in HEK293T cells and immunofluorescence (IF) analysis demonstrated the existence of the interaction between the two proteins (Fig. 3G). Further IF analysis in A2780 and SKOV3 cells confirmed the interaction between endogenous PINK1 and PTEN in ovarian cancer cells (Fig. 3H).

To identify the specific region(s) in PTEN responsible for its interaction with PINK1, four PTEN mutants deleting different domains were constructed (Fig. 3I). HEK293T cells were transfected with Flag-PINK1 and either full-length HA-PTEN or PTEN truncation mutants. After performing co-IP with anti-HA antibody and agarose, Flag-PINK1 was detected by western blot (Fig. 3J, Fig. S4H). We observed that PTEN mutants lacking the PBD domain failed to interact with PINK1 (lane 4 compared to lane 2), highlighting the critical role of the PBD domain in the PINK1-PTEN interaction. Furthermore, molecular simulations provide additional support for the interaction between PINK1 and PTEN (Fig. 3K, L, Fig. S4I).

Together, our findings demonstrate that PTEN is an interaction partner of PINK1, and the PBD domain of PTEN plays a significant role in the interaction.

### PINK1 phosphorylates PTEN at residue Ser179

**Fig. 4:**
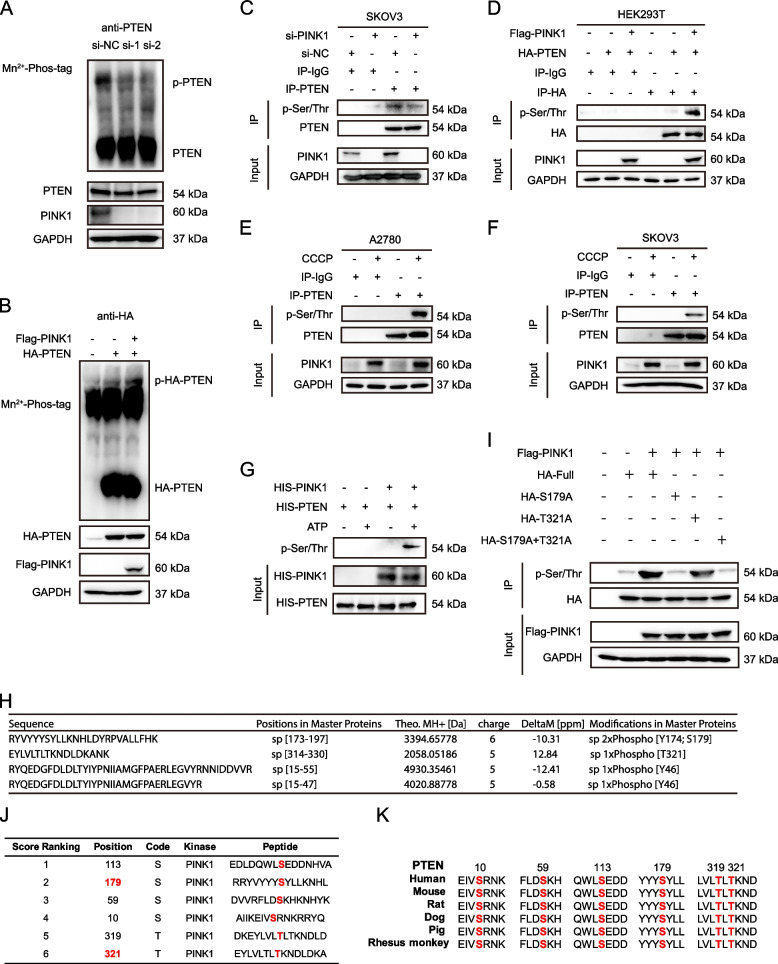
PINK1 phosphorylates PTEN at residue Ser179. **A** Phosphorylation of endogenous PTEN was detected through phos-tag gels upon the knockdown of PINK1 in SKOV3 cells. The lysates were subjected to SDS-PAGE containing Mn^2+^–Phos-tag, followed by immunoblotting with the anti-PTEN antibody. **B** Phosphorylation of exogenous PTEN was detected through phos-tag gels with or without the over-expression of Flag-PINK1 and HA-PTEN in HEK293T cells. The lysates were treated as above and followed by immunoblotting with the anti-HA antibody. **C** PTEN was immunopurified from SKOV3 cells with or without PINK1 knockdown. The phosphorylation on serine/threonine of PTEN was detected by immunoblotting with the anti-pan Phospho-Serine/Threonine (pan p-Ser/Thr) primary antibody. **D** PTEN was immunopurified from HEK293T cells with or without over-expression of Flag-PINK1 and HA-PTEN. The Phosphorylation on serine/threonine of PTEN protein was detected by immunoblotting with the anti-pan p-Ser/Thr primary antibody. **E** and **F** PTEN was immunopurified from A2780 (E) or SKOV3 (F) cells with or without treatment of 10μM CCCP for 6 h. The phosphorylation on serine/threonine of PTEN protein was detected by immunoblotting with the anti-pan p-Ser/Thr primary antibody. **G** In vitro kinase assay. The active kinase, PINK1, and the substrate PTEN, in the presence or absence of adenosine 5’-triphosphate (ATP), were used for kinase assay. Anti-pan p-Ser/Thr primary antibody was applied for detecting phospho-serine/threonine. **H** Phosphorylated residues in PTEN detected by mass spectrometry analysis. **I** Phosphorylation on serine/threonine of PTEN protein immunopurified from HEK293T cells with PTEN mutant over-expression was detected through western blot, immunoblotting with the anti-pan p-Ser/Thr antibody. **J** Phosphorylation sites predicted by GPS 6.0. **K** Sequence conservation analysis of relevant amino acids of PTEN

Given that PINK1 is a serine/threonine protein kinase, we investigated whether PTEN is a substrate for PINK1-mediated phosphorylation. Firstly, SKOV3 cells were transfected with si-PINK1 for 48 h. Then phos-tag gels were applied to separate phosphorylated PTEN proteins. We detected less shifting phosphorylated PTEN when PINK1 was down-regulated (Fig. 4A). In addition, overexpression of HA-PTEN alone or with Flag-PINK1 in HEK293T cells showed increased phosphorylation of PTEN with PINK1 overexpression (Fig. 4B). To confirm that PTEN is phosphorylated at serine/threonine residues, we conducted an immunoprecipitation assay using anti-PTEN antibody and detected serine/threonine phosphorylation of endogenous PTEN, which decreased when PINK1 was knocked down (Fig. 4C). Conversely, overexpression of exogenous PINK1 in HEK293T cells led to an increase in serine/threonine phosphorylation level of HA-PTEN (Fig. 4D). To explore the effect of activated PINK1 on PTEN, we used CCCP, a mitophagy inducer, known to activate PINK1 [45]. Serine/threonine phosphorylation of PTEN increased in A2780 and SKOV3 cells upon application of CCCP (Fig. 4E, F). Finally, in vitro kinase assay showed that PINK1 could phosphorylate PTEN in the presence of ATP (Fig. 4G).

To identify the phosphorylation site of PTEN by PINK1, we conducted mass spectrometry (MS) analysis. Our findings suggest that threonine 321 (Thr321) and serine 179 (Ser179) are probably the residues of PTEN phosphorylated by PINK1 (Fig. 4H, Fig. S4J, K). To verify this, we constructed the corresponding PTEN mutant and detected the serine/threonine phosphorylation levels. We found that mutagenesis of Ser179 significantly abolished the signal of phosphorylation (Fig. 4I), indicating that Ser179 is the primary site for PINK1 phosphorylation of PTEN. This result aligns with our MS data, as the abundance of phospho-Ser179-containing peptides outmatched those of phospho-Thr321 (Table S4). Moreover, GPS 6.0 was used to predict the PTEN phosphorylation site(s) by PINK1 (Fig. 4J) [46, 47]. Consistent with MS results, Ser179 was predicted with high scores. Furthermore, Ser179 was highly conserved across most PTEN proteins from different species (Fig. 4K).

### PINK1 enhances the activity of AKT by regulating PTEN

**Fig. 5:**
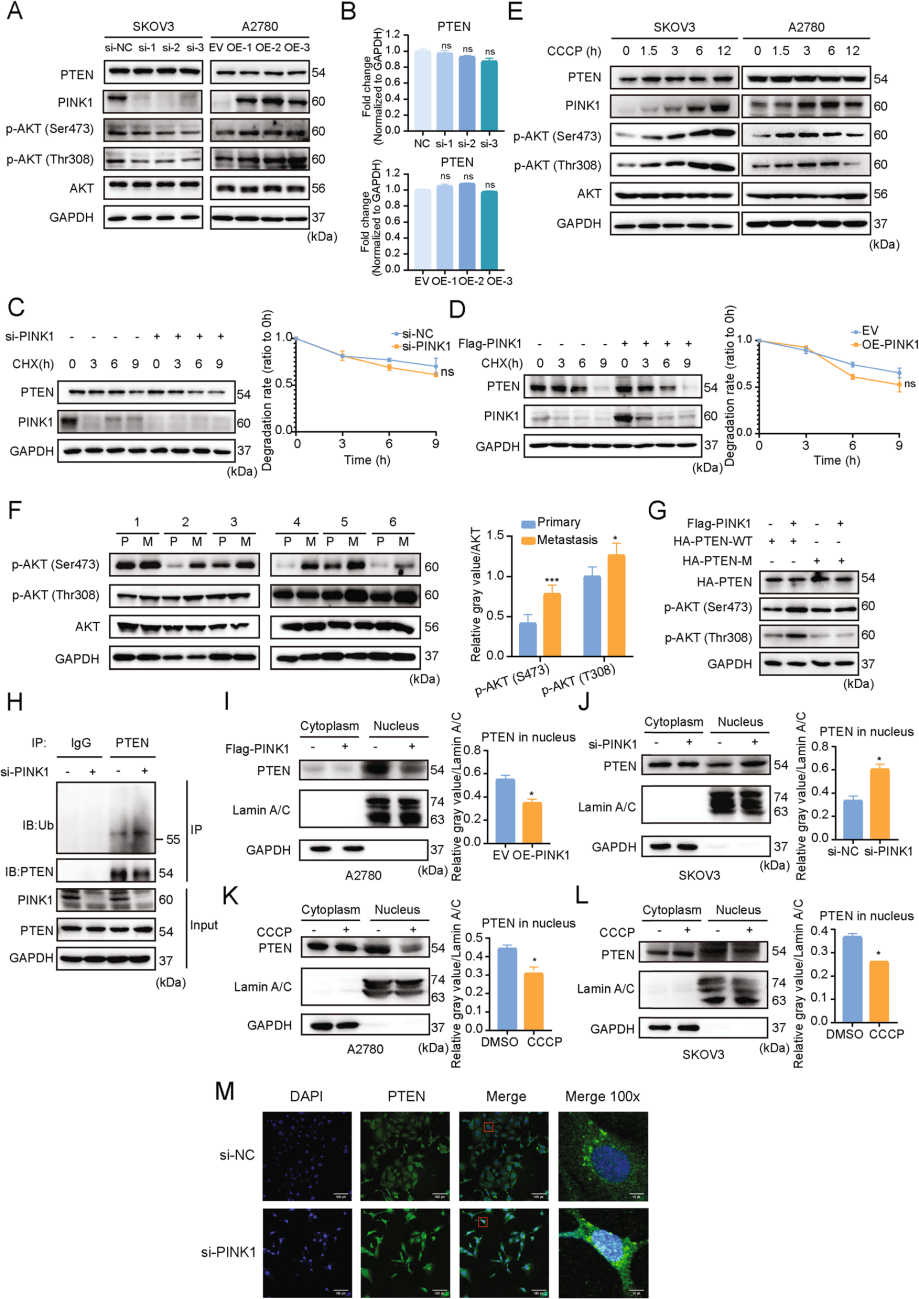
PINK1 inhibits PTEN nuclear import and activates AKT via phosphorylating PTEN. **A** SKOV3 and A2780 cells were transfected with PINK1-targeting siRNA or PINK1 over-expression plasmid. Western blot analysis was conducted to evaluate the protein of PTEN, AKT, p-AKT (T308) and p-AKT (S473). **B** SKOV3 and A2780 cells were transfected with PINK1-targeting siRNA or PINK1 over-expression plasmid. qRT-PCR analysis was conducted to evaluate the mRNA level of PTEN. **C **and** D**, Time-course analysis of PTEN protein levels in SKOV3 (**C**) and A2780 (**D**) cells, which were transfected with PINK1-targeting siRNA or PINK1 over-expression vector respectively. PTEN protein level were quantified and plotted on the right. **E**, SKOV3 and A2780 cells were treated with CCCP for indicated duration. Western blot analysis were conducted to evaluate the protein levels of PTEN, AKT, p-AKT (T308) and p-AKT (S473). **F**, The expression level of p-AKT in paired ovarian cancer tissues. The protein level was quantified and plotted on the right. **G**, The expression level of p-AKT in HEK293T cells transfected with PTEN mutant (S179A) and WT over-expression plasmid. **H** SKOV3 cells were transfected with PINK1 targeting siRNA (si-PINK1 +) or negative control siRNA (si-PINK1 -), followed by immunoprecipitation with anti-PTEN. The protein level of ubiquitin and PTEN was analyzed by western blot. **I **and** J**, A2780 (**I**) and SKOV3 (**J**) cells were transfected with PINK1 over-expressing plasmid or siPINK1 respectively, followed by nucleocytoplasmic separation. The protein level of PTEN was analyzed by western blot and the quantification of PTEN protein level in nucleus was shown. **K** and **L**, A2780 (**K**) and SKOV3 (**L**) cells were exposed to CCCP (10μM) for 6 h, followed by nucleocytoplasmic separation. The protein level of PTEN was analyzed by western blot and the quantification of PTEN protein level in nucleus was shown. **M** The subcellular localization of PTEN in SKOV3 cells transfected with siPINK1 or siNC. The transfected cells were visualized using an antibody against PTEN. ns, no statistical significance. * *p* < 0.05, *** *p* < 0.001. Values are mean ± SEM. Data are representative of 3 independent experiments

To investigate the potential role of PINK1 in regulating PTEN, we over-expressed PINK1 in A2780 cells and knocked down PINK1 in SKOV3 cells. However, the expression of neither PTEN mRNA nor protein was affected by either manipulation (Fig. 5A, B). Furthermore, time-course experiments confirmed that the protein stability of PTEN was not influenced by changes in PINK1 expression (Fig. 5C, D). The tumor suppressive effect of PTEN is mainly through the inhibition of PI3K/AKT signaling[8], and previous studies have shown that the functional inactivation of PTEN may be related to its phosphorylation [48, 49]. Therefore, we detected the expression levels of p-AKT. Notably, knocking down PINK1 led to a decreased expression of p-AKT (Thr308, Ser473), suggesting that PINK1 interacts with PTEN to activate AKT (Fig. 5A, Fig. S5A). Supporting this notion, we observed that treatment with a mitophagy inducer led to increased p-AKT levels in a time-dependent manner(Fig. 5E, Fig. S5B). Moreover, the protein level of p-AKT was higher in human ovarian cancer metastases tissues compared to primary tumors (Fig. 5F), consistent with the expression level of PINK1 (Fig. 1H).

To determine whether PINK1-mediated PTEN phosphorylation affected the phosphorylation level of AKT, we overexpressed a PTEN mutant with a mutation at Ser179 and detected p-AKT protein level. We found that overexpression of PTEN mutant, together with PINK1, failed to enhance the level of p-AKT, as compared to the wildtype group (Fig. 5G, lane 3 and 4).

Collectively, our data indicate that PINK1 phosphorylates PTEN at Ser179, leading to activation of AKT and promotion of tumor metastasis and cell survival.

### PINK1 inhibits PTEN nuclear translocation

Previous studies have shown that phosphorylating PTEN may result in ubiquitination, leading to an impact on its function and destiny [28]. Therefore, PINK1 was silenced in SKOV3 cells to explore the ubiquitination level of PTEN. As a result of PINK1 knockdown, the level of monoubiquitination of PTEN increased (Fig. 5H). Studies have suggested that the monoubiquitination level of PTEN has the potential to implicate its tumor suppressor function by regulating its nuclear transport [22]. Therefore we examined whether phosphorylation by PINK1 could affect the nuclear transport of PTEN, thereby potentially promoting cancer metastasis and chemoresistance. Following the overexpression or knockdown of PINK1, we carried out nucleoplasmic separation on ovarian cancer cells. It was observed that after PINK1 overexpression, the PTEN level in the nucleus decreased, while it increased after PINK1 knockdown (Fig. 5I, J). PTEN levels in the cytoplasm were not significantly altered (Fig. 5I, J, Fig. S5C, D). To investigate if PINK1 activation via the induction of mitophagy could have the same effect, we treated cells with CCCP followed by nucleoplasmic separation. Consistent with these results, the level of PTEN into the nucleus decreased significantly after PINK1 activation (Fig. 5K, L, Fig. S5E, F). To visualize the location of PTEN in response to phosphorylation by PINK1, immunofluorescence experiments were performed and we observed that the PTEN (green) puncta within the nucleus apparently increased after PINK1 knockdown (Fig. 5M).

Collectively, these results indicate that PINK1 reduces the mono-ubiquitination of PTEN by phosphorylating it at Ser179, inhibiting the nuclear translocation of PTEN. This effect, in turn, activates AKT, promoting the metastasis and chemotherapy resistance of ovarian cancer.

### PINK1 promotes the metastasis and cisplatin resistance of ovarian cancer in vivo

**Fig. 6:**
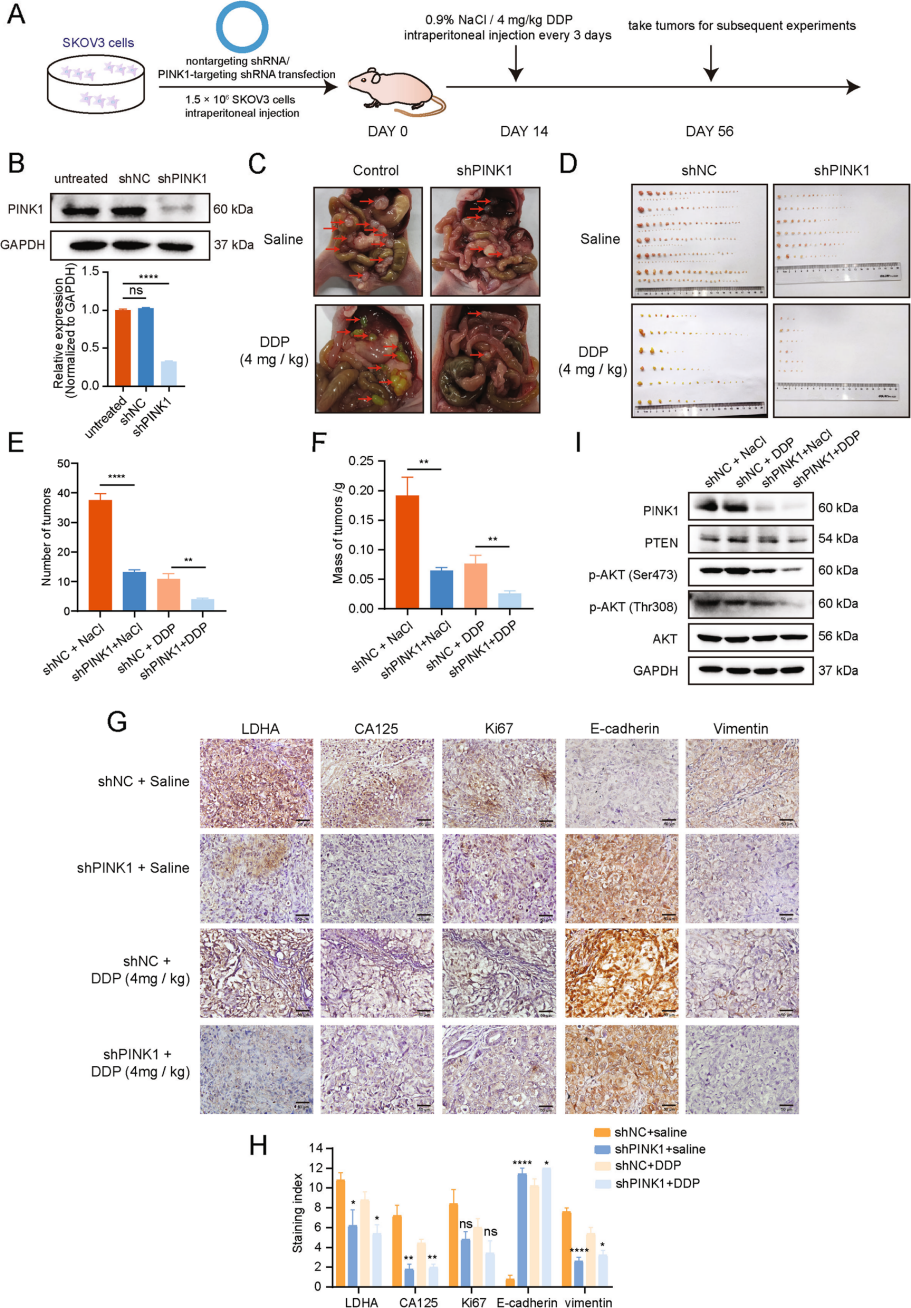
PINK1 promotes the metastasis of ovarian cancer and cisplatin resistance via regulating PTEN. **A** Schematic diagram of animal experiment procedure. **B** Knock down efficiency of shRNA targeting PINK1 evaluated by western blot and qRT-PCR. **C** Representative images of metastatic nodules in the abdomens of nude mice injected with SKOV3 cells stably expressing an empty vector or PINK1-targeting shRNAs, and administered DDP. **D** The metastatic tumors were separated and recorded. **E** and **F** Quantitative analysis of the number (**E**) and weight (**F**) of metastatic tumors in mice. **G** and** H** Representative images (**G**) and quantitative analysis (**H**) of IHC staining of CA125, Ki67, LDHA, E-cadherin and vimentin proteins. **I** Western blot detection of indicated protein expression levels in metastatic nodules located in the abdomens of nude mice subjected to specific treatments. * *p* < 0.05, ** *p* < 0.01, and **** *p* < 0.0001. Values are mean ± SEM. Data are representative of 3 independent experiments

To evaluate the effect of PINK1 on cancer metastasis and drug resistance, xenograft models in vivo was applied. Intraperitoneally injections of SKOV3 cells expressing PINK1-targetting shRNA (shPINK1) or negative control shRNA (shNC) into BALB/c nude mice were carried out, and they received DDP or saline every 3 days (Fig. 6A). The knockdown efficiency of PINK1 was shown in Fig. 6B. Eight weeks after cell injection, the cancer cells transfected with shPINK1 formed fewer nodules than the other groups (Fig. 6C-E). In addition, we found that the group receiving intraperitoneal injections of shPINK1-transfected tumor cells alongside DDP had the smallest mass and volume of metastatic tumor(Fig. 6C-F).

Moreover, we assessed the malignancy markers, including LDHA, CA125, and Ki67 in ovarian cancer. The representative IHC images showed weaker positive indicators in shPINK1 groups as compared to the control group, depicting significant differences (Fig. 6G, H). To evaluate the direct influence of PINK1 on the EMT in ovarian cancer, IHC assay for EMT markers (E-cadherin and vimentin) was performed, which indicated a decreased level of vimentin and an increased level of E-cadherin upon PINK1 knockdown (Fig. 6G, H). Based on the results, the knockdown of PINK1 could enhance the effectiveness of cisplatin treatment as well as reduce the metastatic potential of tumors. Additionally, PI3K/PTEN/AKT markers were detected for the nodules, and the results were consistent with those observed in vitro (Fig. 6I).

Taken together, our findings demonstrate that PINK1 regulates the tumor suppressor function of PTEN through its phosphorylation, resulting in promoting tumor metastasis and chemotherapy resistance.

## Discussion

Ovarian cancer represents one of the deadliest among all gynecologic malignancies following breast cancer [50]. Tumor metastasis and chemotherapy resistance are major factors leading to a poor prognosis of ovarian cancer. Autophagy has been shown to play a crucial role in cancer metastasis [51] and drug resistance [52] in recent years. In this paper, we identified PINK1 from autophagy-related kinases as the most closely associated kinase with poor prognosis of ovarian tumor. Since Parkin expression is extremely low in ovarian cancer, we focused on exploring the function of PINK1 in the absence of Parkin. Finally, we demonstrated that the kinase PINK1 could phosphorylate PTEN at Ser179, inducing defects in the nuclear translocation and phosphatase activities of PTEN. This phosphorylation prevents PTEN from reducing the activity of the AKT signaling pathway, leading to the higher p-AKT levels and greater metastasis ability. Furthermore, the use of in vivo xenograft models revealed that PINK1 knockdown could inhibit abdominal metastasis and chemotherapy resistance of ovarian cancer. Therefore, our current study proposes new insights into a novel mechanism of ovarian cancer metastasis and chemotherapy resistance, and provides a theoretical basis of possible clinical treatment through PINK1 inhibition.

As mentioned before, PINK1 is a putative serine/threonine kinase, initially discovered as a gene governed by the tumor suppressor PTEN in cancer cells [9]. The major progress of the research on PINK1 was to better understand Parkinson disease [53]. Subsequently, PINK1 was reported to be high expression in mouse cancer cells of melanoma and colon carcinoma, which displayed significant metastatic potential [50]. Later on, mounting evidence indicates that PINK1 is implicated in diverse aspects of tumor biology.

While plenty of researched have identified PINK1 as an important player in mitochondria, there is growing evidence indicating that PINK1 activity is not limited to mitophagy. Diverse subcellular PINK1 are involved in different signaling cascades to regulate tumor occurrence and development.

Our findings revealed a correlation between PINK1 and a negative prognosis for ovarian cancer patients based on open database and our own local cohort (Fig. 1). The hazard ratio of PINK1, which seems weak, may resulted from the including of all the datasets with no restriction. Different datasets contains patients with diverse clinicopathological features, such as varying FIGO stage, histological subtypes, treatment, and metastasis condition. In addition,the inclusion of diverse races, age distribution and sample size may also attenuate the positive association.

Considering the comparable HR value of PINK1 and WIPI1, we used Kaplan–Meier Plotter database to further evaluate the association between PINK1 (or WIPI1) expression level and overall survival in patients with ovarian cancer. The results showed that the high expression level of PINK1 was significantly associated with the worse prognosis in ovarian cancer patients (Fig. 1C), while the WIPI1 expression level was not significant related to the poor prognosis (Fig. S1B). The results suggested that the association between PINK1 and poor prognosis of ovarian cancer was more stable and convincing, so PINK1 was chosen for further research. Nevertheless, the effect of WIPI1 could not be ruled out and it may play an important role in some particular histological types of ovarian cancer. We are also interested in doing research relating to WIPI1 in the future.

Tumors develop in a complex microenvironment and maintain the growth, invasion and metastasis relying on this intricate milieu. The intrinsic characteristics of tumor cells, as well as the microenvironment, exert significant influence on tumor growth and metastasis [54–57]. The metastasis of tumor cells is a multiple process that plays a vital role in the cancer-related morbidity and mortality. For ovarian cancer, the omentum represents the most common site of metastasis [4]. One of the reasons for the preferential localization of cancer cells to the omentum is the absence of the basement membrane and the lack of mesothelial cells on the surface of milky spots. These conditions facilitate the invasion of cancer cells [4, 58]. In our study, we found that PINK1 was significantly associated with the omental metastasis in ovarian cancer, while no significant correlation had been observed with lymph node metastasis or metastasis to other organs (Fig. 1G). This suggests that PINK1 may play an important role in the specific metastasis process of ovarian cancer to the omentum. However, we do not exclude the possibility of a correlation between PINK1 and metastasis to other sites in ovarian cancer, as the positive correlation may be masked due to the limited number of patients in the cohort. Furthermore, the different histological types of ovarian cancer and their interactions with the microenvirenment may also influence the tendency and sites of metastasis. The potential correlation between PINK1 and the tendency for ovarian cancer metastasis warrants further investigation.

Previous studies have reported a consistency with our own findings, which further demonstrated that a lower expression level of PINK1 was linked to a significantly prolonged overall survival in patients with nonsmall-cell lung cancer (NSCLC) and esophageal squamous cell carcinoma (ESCC) [59]. Similarly, several analyses in NSCLC patients revealed that high expression levels of PINK1 were associated with a compromised treatment response and were identified as an independent prognostic factor for adenocarcinoma [60, 61]. Additionally, a meta-analysis conducted on human ovarian cancer patients indicated that a high PINK1 mRNA expression is correlated with a negative prognosis [13]. Cell-based analysis has shown that PINK1 inhibition increases apoptosis rate and decreases metastatic abilities across multiple lung cancer cell lines [60, 62], which is consistent with our results in ovarian cancer cell lines (Fig. 2A, B, Fig. S2G, H). In breast cancer, PINK1 could drive the production of extracellular vesicles containing Mitochondrial DNA (mtDNA), which promote invasiveness [63]. Moreover, various studies have reported PINK1’s critical role in cancer cell survival and resistance to chemotherapy, therefore indicating it as a potential therapeutic target for tumor treatment [11, 64, 65]. This data suggests that PINK1 might be a potent cancer-promoting kinase.

In our experimental setting, we demonstrated that, in ovarian cancer, PINK1 played a critical role in promoting metastasis and chemotherapy resistance (Figs. 1 and 2). PINK1 promotes tumor cell invasion and migration depending on its kinase activity (Fig. 2A, B). Our results also encourage the relevance of PINK1 expression levels with ovarian cancer prognosis (Fig. 1). In principle, these data argue a prominent role of PINK1 in mediating metastasis and chemoresistance of ovarian cancer.

Most of its essential functions of PINK1 are performed by phosphorylating its downstream substrates as a kinase. The most canonical substrate is Parkin since PINK1 could activate mitophagy by phosphorylating ubiquitin and recruiting Parkin [17, 66, 67]. However, plenty of studies have found that PINK1 could carry out its biological regulatory function independently of Parkin. Liu et al. [18] reported that PINK1 was able to phosphorylate and activate p53, disturbing its nuclear localization and the function of stemness inhibition. Arena et al. [68] found that PINK1 could interact with Bcl-xl and phosphorylate it upon mitochondrial depolarization, regulating cell survival in Parkinson’s disease and cancer. PINK1 was also shown to phosphorylate mitofusin for the regulation of mitochondrial fusion [69]. Lazarou et al. [15] revealed that PINK1 could recruit NDP52 and optineurin to induce mitophagy directly, outside of Parkin involvement.

In addition, our results revealed that Parkin expression level was extremely low in ovarian cancer, indicating that PINK1 regulates other substrates to promote ovarian cancer metastasis and chemoresistance. Considering that PINK1 was originally identified as a gene that is up-regulated by PTEN, we investigate whether PTEN could be interacted and regulated by PINK1 to form a loop. In the present study, we found that PINK1 could interact with and phosphorylate PTEN at Ser179 (Figs. 3 and 4), resulting in reduction of activity and nuclear translocation of PTEN (Fig. 5).

PTEN is an important tumor suppressor and governs various biological processes, such as cell survival, migration, and invasion [22]. Therefore, the regulation of PTEN has become an intense research topic in tumor biology. Phosphorylation is a vital post-translational modifications that regulates the function and activity of PTEN. 32 phosphorylation sites on PTEN has been revealed in PhosphoSitePLus (https://www.phosphosite.org/) identified by mass spectrometry analysis or/and other methods. Phosphorylation of PTEN at different sites will affect its diverse functions, including activity, protein stability and cellular localization. Studies have shown that PTEN phosphorylation mediated by CK2 influences its activity and protein stability by preventing degradation [28, 70]. It is worth noting that the effects of PTEN phosphorylation are subject to variation based on different cytological types and conditions. CK2-mediated phosphorylation of PTEN in T-ALL cells leads to the down-regulated activity, thereby promoting PI3K/AKT constitutive hyperactivation [49]. And the phosphorylation of PTEN on Thr366 results in destabilization rather than stabilization in glioma cells [26]. In addition, phosphorylation of PTEN by ATM has been revealed to effect its nuclear translocation [71]. Here we identified the phosphorylation at Ser179 of PTEN by PINK1 through mass spectrometry and in vitro experiments. This phosphorylation site has been first identified by mass spectrometry in melanoma skin cancer tissues and Jurkat cell lines in 2009, reported on PhosphoSitePLus.

It has been revealed that PTEN inactivation induces metastasis in non-small cell lung carcinoma [72]. Similarly, Yang et al. reported that hepatoma-derived exosomal miR92a-3p could inhibit PTEN and activate AKT/Snail signaling to promote metastasis and EMT [73]. Song et al. found that silencing TULP3 suppresses gastric tumor cell metastasis through PTEN/AKT/Snail pathway [74]. Patsoukis et al. determined that PD-1 regulated the lipid phosphatase activity of PTEN and inhibited PI3K/AKT pathway by suppressing CK2 activity [24]. Our results revealed that PINK1 facilitate metastasis and chemoresistance by phosphorylating PTEN and activating AKT independent of Parkin.

The interaction between PINK1 and PI3K-AKT has also been studied extensively in cancer. Previous studies have shown that PINK1 can directly activate AKT via activation of mTORC2 to enhance invasiveness of cancer cells [75]. In HeLa cells, PINK1 was found to induce AKT phosphorylation of hexokinase-II at the mitochondria [76]. In Parkinson's disease, PINK1 was found to be a primary upstream activator of Akt via regulation of PIP3 [77]. Here we found that PINK1 could activate AKT via the phosphorylation and reduction activity of PTEN. The regulatory mechanisms between PINK1 and AKT is worth further study.

Earlier studies pay more attention to the function of PTEN in cytoplasm. Recent studies have suggested that nuclear PTEN complemented with cytoplasmic PTEN to inhibit cancer development. Ubiquitination, SUMOylation and phosphorylation have been shown to regulate the shuttling of PTEN between the cytoplasm and the nucleus [71, 78, 79]. Poly-ubiquitination of PTEN usually regulates its stability while mono-ubiquitination has been shown to control its nuclear localization [71, 78, 80]. Here we demonstrated that the phosphorylation at Ser179 of PTEN by PINK1 inhibits the mono-ubiquitination and nuclear translocation of PTEN, therefore the antitumor effect of PTEN was diminished. Nuclear PTEN plays essential roles including its lipid phosphatase activity in inhibiting tumor development [81]. Previous studies have reported the tumor suppressor p53 has an increased stability and transcriptional activity due to the action of PTEN [82]. Xie et al. have reported that PTEN neddylation regulated its nuclear import and promoted the progression of breast tumor [83]. Yu et al. found that the inhibition of nuclear translocation of PTEN by Myosin 1b could regulate the activation of nuclear AKT [84]. This study revealed that phosphorylation of PTEN at Ser179 by PINK1 prevented nuclear import, thus promoting ovarian cancer metastasis and cisplatin resistance. In xenograft models, we found that PINK1 inhibition can improve the chemotherapy effect of cisplatin and reduce the ability of tumor metastasis. In our future projects, genetic animal models [85, 86], patient-derived xenografts models, and organoid models, may help to further understanding the impact of PINK1-PTEN axis on ovarian cancer and determine more effective treatments for ovarian cancer patients. PINK1 knock-out mice [87] could be applied to construct new ovarian cancer models. In addition, consdering the PINK1 is a putative kinase, targeting PINK1 through screening specific kinase inhibitors may be a promising way [88–90]. The population intervention of PINK1 in the future may be a valuable approach for prevention and treatment of ovarian cancer. In summary, inhibition of PINK1 by potential drug may be a promising candidate for ovarian cancer treatment.

## Conclusions

**Fig. 7:**
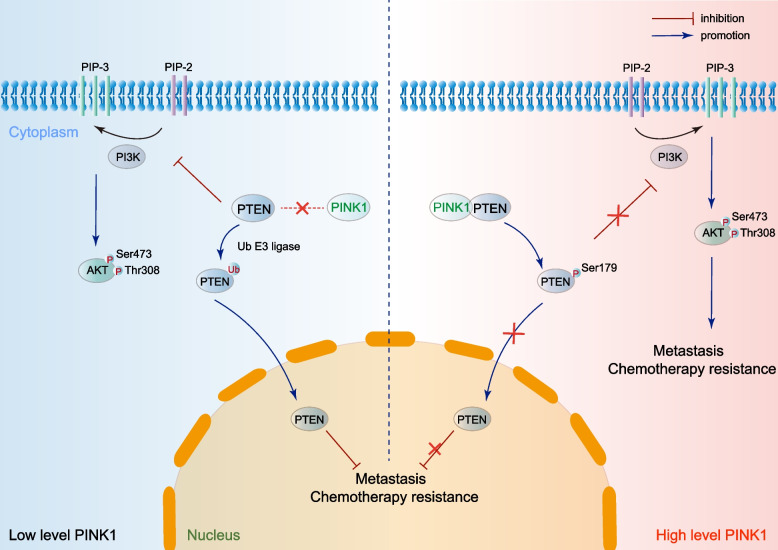
PINK1-PTEN axis promotes ovarian cancer metastasis and chemoresistance independent of Parkin. PINK1 interacts with and phosphorylates PTEN at Ser179 in ovarian cancer, leading to increased p-AKT levels and inhibition of PTEN nuclear import. This, in turn, promotes metastasis and chemoresistance. Inhibiting PINK1 may be an effective method for restraining metastasis and chemoresistance in ovarian cancer

In conclusion, our study identified PINK1-PTEN axis promotes ovarian cancer through non-canonical pathway independent of Parkin (Fig. 7). PINK1 could up-regulate the activity of AKT and inhibit PTEN nuclear translocation by phosphorylating PTEN at Ser179, which eventually promote the metastasis and chemotherapy resistance of ovarian cancer. Therefore, PINK1 might be a novel therapeutic target for ovarian cancer treatment.

### Supplementary Information

**Additional file 1:** **Figure S1.** A Forest plot of Hazard Ratios calculating from the “CuratedOvarianData”. B Forest plot of Hazard Ratios for evaluating the association between WIPI1 expression and overall survival of ovarian cancer patients. The data included was obtained from Kaplan-Meier website and was stratified based on WIPI1 expression. The red dotted line represents the pooled HR of meta-analysis. C The correlation between PINK1 expression and tumor diameter. **Figure S2.** A and B Western blot analysis for protein levels of PINK1 in five ovarian related cell lines (A) and quantitative statistics (B). C Expression of PINK1 mRNA in ovarian cancer cell lines downloaded from The Human Protein Atlas. D Expression levels of PINK1 mRNA in five ovarian related cell lines. E Validation of siRNA knockdown efficiency in SKOV3 cells as determined by western blot (left) and qRT-PCR (right). F Validation of the efficiency of Flag-PINK1 over-expression vector in SKOV3, A2780 and HEK293T cells as determined by western blot. EV, expression vector. OE, over-expression. M, inactive mutant. G Cells were transfected with PINK1-target siRNA for 24 h. Representative images of the wound scratch assay utilizing the SKOV3 cell lines after scratching 24 and 48 h. The histograms on the right show the quantitative results of the healing percentage after 48 h of three independent replicates. H Effects of PINK1 on SKOV3 cell invasion (upper) and migration (bottom). Cells were transfected by PINK1-target siRNA for 24 h, and then treated as Figure 2B. **Figure S3.** A Representative images (left) and quantitative analysis (right) of proliferating SKOV3 cells, which were transfected by siRNA targeting PINK1 for 48 h and then assessed by EdU kit assay. B and C Effects of PINK1 on proliferation ability of A2780 and SKOV3 cells. A2780 (B) and SKOV3 (C) cells were transfected by PINK1 over-expression plasmid and siRNA targeting PINK1 respectively for 24, 48, and 72 h, and then assessed by CCK-8 kit assay. D Western blot analysis of EMT markers expression. SKOV3 cells were transfected by PINK1-targeting siRNA. E and F An analysis of Overall Survival (E) and Progression-Free Survival (F) curves in Stage 3+4 human ovarian cancer, utilizing data available on the Kaplan-Meier Plot website. All relevant data on the website were stratified according to PINK1 expression. ns, no statistical significance. Values are mean ± SEM. Data are representative of 3 independent experiments. **Figure S4.** A The protein expression level of PRKN in diverse tissues downloaded from The Human Protein Atlas. B PTEN was detected by mass spectrometry analysis. C Interaction prediction of PINK1 and PTEN by genemania database. D Interaction prediction of PINK1 and PTEN by string database. E Interaction prediction of PINK1 and PTEN by hitpredict analysis. F and G Western blot analysis of endogenous PINK1 and PTEN expression in a co-IP assay performed in SW480 cells with Protein A/G PLUS-Agarose and anti-PINK1 (F) or anti-PTEN (G) primary antibody. H Quantification analysis of PINK1 interacted with PTEN in Figure 3J. I The Z-DOCK score of PINK1 and PTEN interaction simulation models. J and K Phosphorylated Ser179 (J) and Thr321 (K) residue in PTEN detected by mass spectrometry analysis of SKOV3 cells. **Figure S5.** A Quantification of p-AKT in Figure 5A. B Western blot analysis of p-AKT in A2780 and SKOV3 cells treated with oligomycin and antimycin (OA), a mitophagy inducer, for indicated time. C and D Quantification of PTEN protein level in cytoplasm in Figure 5I and J. E and F Quantification of PTEN protein level in cytoplasm in Figure 5K and L.

**Additional file 2:** **Table S1.** The detailed antibody information. **Table S2.** Primers and sequences. **Table S3.** Detailed information of included datasets from the Kaplan-Meier Plotter. **Table S4.** The detailed mass spectrometry information for peptidegroup.

**Additional file 3.** Supplementary material for methods.

## Data Availability

All data that support the findings of this study is included in the supplementary information or is accessible from the authors upon reasonable request.

## Abbreviations

PINK1: PTEN-induced kinase 1
PTEN: Phosphatase and tensin homolog
PRKN: Parkin RBR E3 ubiquitin protein ligase
ULK1/2: Unc-51 like autophagy activating kinase 1/2
tp53: Tumor protein p53
MFN2: Mitofusin 2
PI3K: Phosphatidylinositol 3-kinases
GSK3β: Glycogen synthase kinase-3 beta
RhoA: Ras homolog family member A
PBD: Phosphatidylinositol (PtdIns) (4,5)P2-binding domain
PBS: Phosphate buffered saline
EdU: 5-Ethynyl-2'-deoxyuridine
IHC: Immunohistochemistry
DDP: Cisplatin
CCK-8: Cell counting Kit-8
IC_50_: Half maximal inhibitory concentration
siRNA: Small interfering RNA
shRNA: Short hairpin RNA
IF: Immunofluorescence
SEM: Standard error of mean
EMT: Epithelial-mesenchymal transition
WT: Wild type
co-IP: Co-immunoprecipitation
MS: Mass spectrometry
