# Supplementary material for: PINK1-PTEN axis promotes metastasis and chemoresistance in ovarian cancer via non-canonical pathway
Source: J Exp Clin Cancer Res. 2023 Nov 9;42:295. doi: 10.1186/s13046-023-02823-w (PMC10633943; doi:10.1186/s13046-023-02823-w)
Supplement: Supplementary file 1 — Additional file 1: Figure S1. A Forest plot of Hazard Ratios calculating from the “CuratedOvarianData”. B Forest plot of Hazard Ratios for evaluating the association between WIPI1 expression and overall survival of ovarian cancer patients. The data included was obtained from Kaplan-Meier website and was stratified based on WIPI1 expression. The red dotted line represents the pooled HR of meta-analysis. C The correlation between PINK1 expression and tumor diameter. Figure S2. A and B Western blot analysis for protein levels of PINK1 in five ovarian related cell lines (A) and quantitative statistics (B). C Expression of PINK1 mRNA in ovarian cancer cell lines downloaded from The Human Protein Atlas. D Expression levels of PINK1 mRNA in five ovarian related cell lines. E Validation of siRNA knockdown efficiency in SKOV3 cells as determined by western blot (left) and qRT-PCR (right). F Validation of the efficiency of Flag-PINK1 over-expression vector in SKOV3, A2780 and HEK293T cells as determined by western blot. EV, expression vector. OE, over-expression. M, inactive mutant. G Cells were transfected with PINK1-target siRNA for 24 h. Representative images of the wound scratch assay utilizing the SKOV3 cell lines after scratching 24 and 48 h. The histograms on the right show the quantitative results of the healing percentage after 48 h of three independent replicates. H Effects of PINK1 on SKOV3 cell invasion (upper) and migration (bottom). Cells were transfected by PINK1-target siRNA for 24 h, and then treated as Figure 2B. Figure S3. A Representative images (left) and quantitative analysis (right) of proliferating SKOV3 cells, which were transfected by siRNA targeting PINK1 for 48 h and then assessed by EdU kit assay. B and C Effects of PINK1 on proliferation ability of A2780 and SKOV3 cells. A2780 (B) and SKOV3 (C) cells were transfected by PINK1 over-expression plasmid and siRNA targeting PINK1 respectively for 24, 48, and 72 h, and then assessed by CCK-8 kit assay. [file 13046_2023_2823_MOESM1_ESM.pdf]

Supplementary Figures

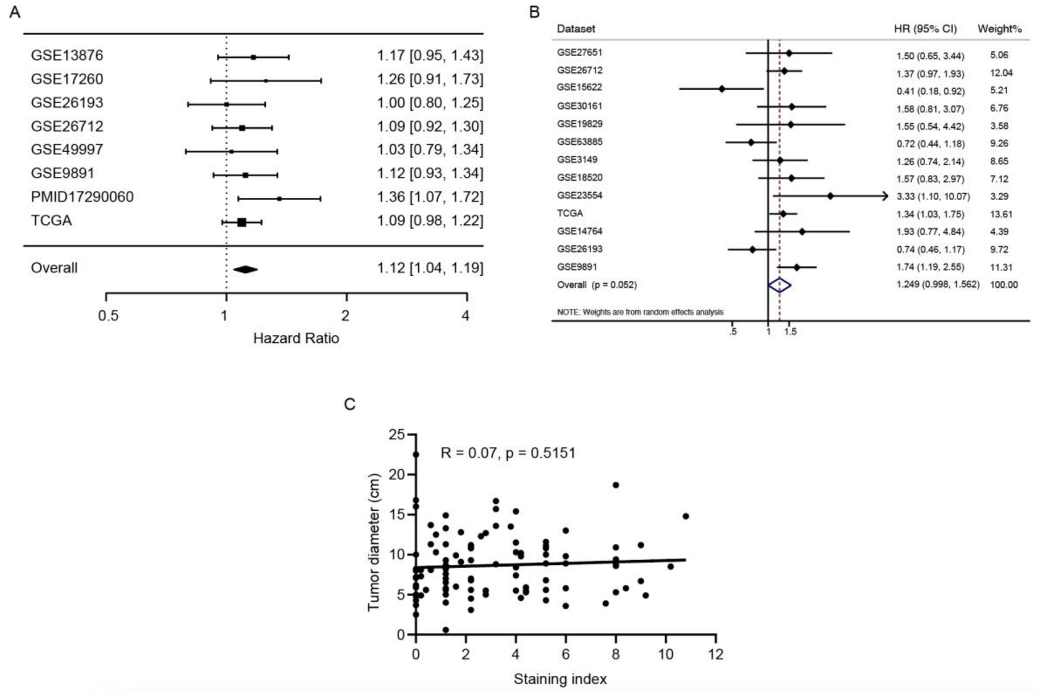

Figure S1

A Forest plot of Hazard Ratios calculating from the “CuratedOvarianData”.

B Forest plot of Hazard Ratios for evaluating the association between WIPI1 expression and overall survival of ovarian cancer patients. The data included was obtained from Kaplan-Meier website and was stratified based on WIPI1 expression. The red dotted line represents the pooled HR of meta-analysis.

C The correlation between PINK1 expression and tumor diameter.

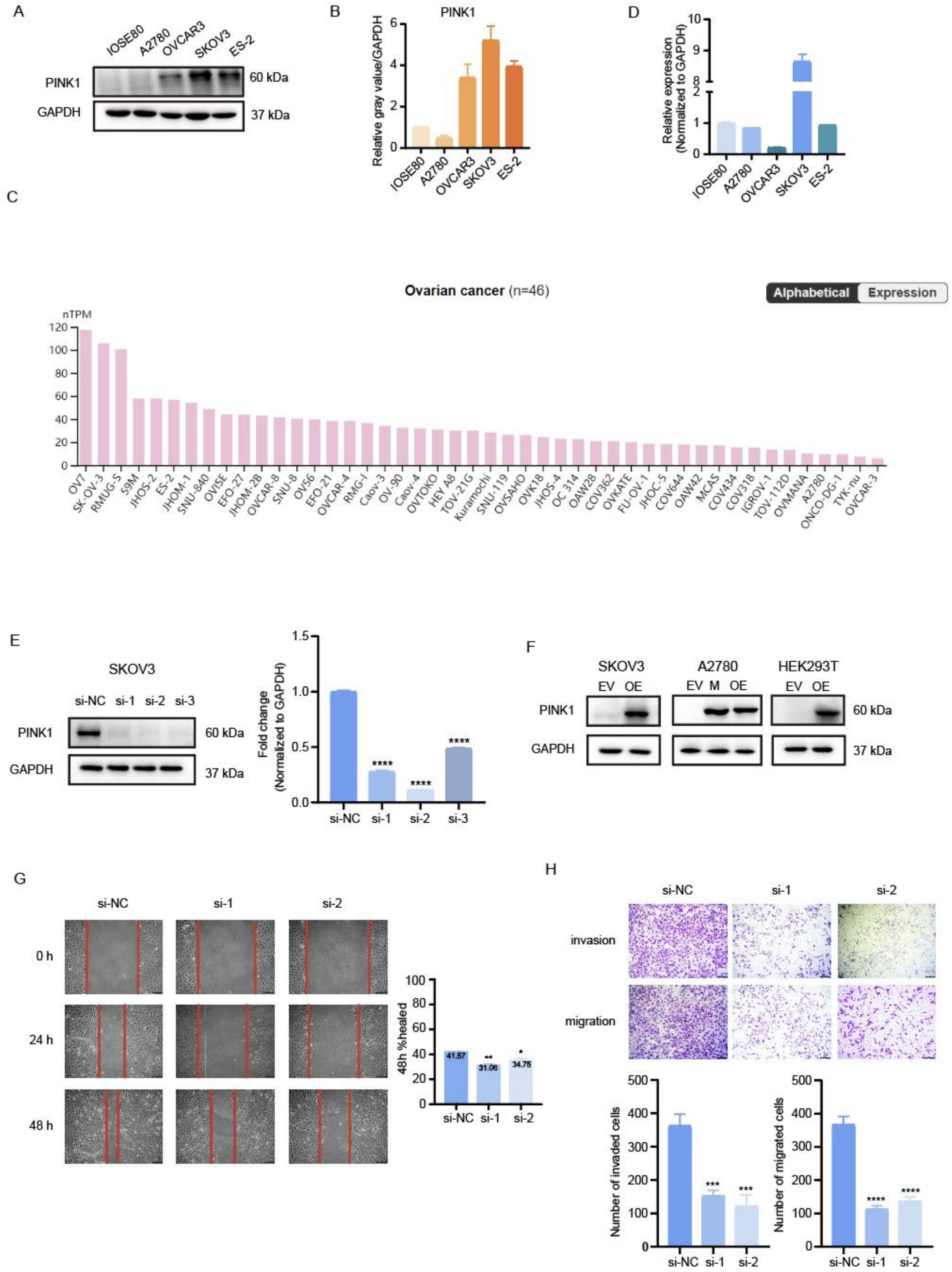

Figure S2

A and B Western blot analysis for protein levels of PINK1 in 5 ovarian related cell lines (**A**) and quantitative statistics (**B**). **C** Expression of PINK1

mRNA in ovarian cancer cell lines downloaded from The Human Protein Atlas. **D** Expression levels of PINK1 mRNA in 5 ovarian related cell lines. **E** Validation of siRNA knockdown efficiency in SKOV3 cells as determined by western blot (left) and qRT-PCR (right). **F** Validation of the efficiency of Flag-PINK1 over-expression vector in SKOV3, A2780 and HEK293T cells as determined by western blot. EV, expression vector. OE, over-expression. M, inactive mutant. **G** Cells were transfected with PINK1-target siRNA for 24 h. Representative images of the wound scratch assay utilizing the SKOV3 cell lines after scratching 24 and 48 h. The histograms on the right show the quantitative results of the healing percentage after 48 h of three independent replicates. **H** Effects of PINK1 on SKOV3 cell invasion (upper) and migration (bottom). Cells were transfected by PINK1-target siRNA for 24 h, and then treated as Figure 2B.

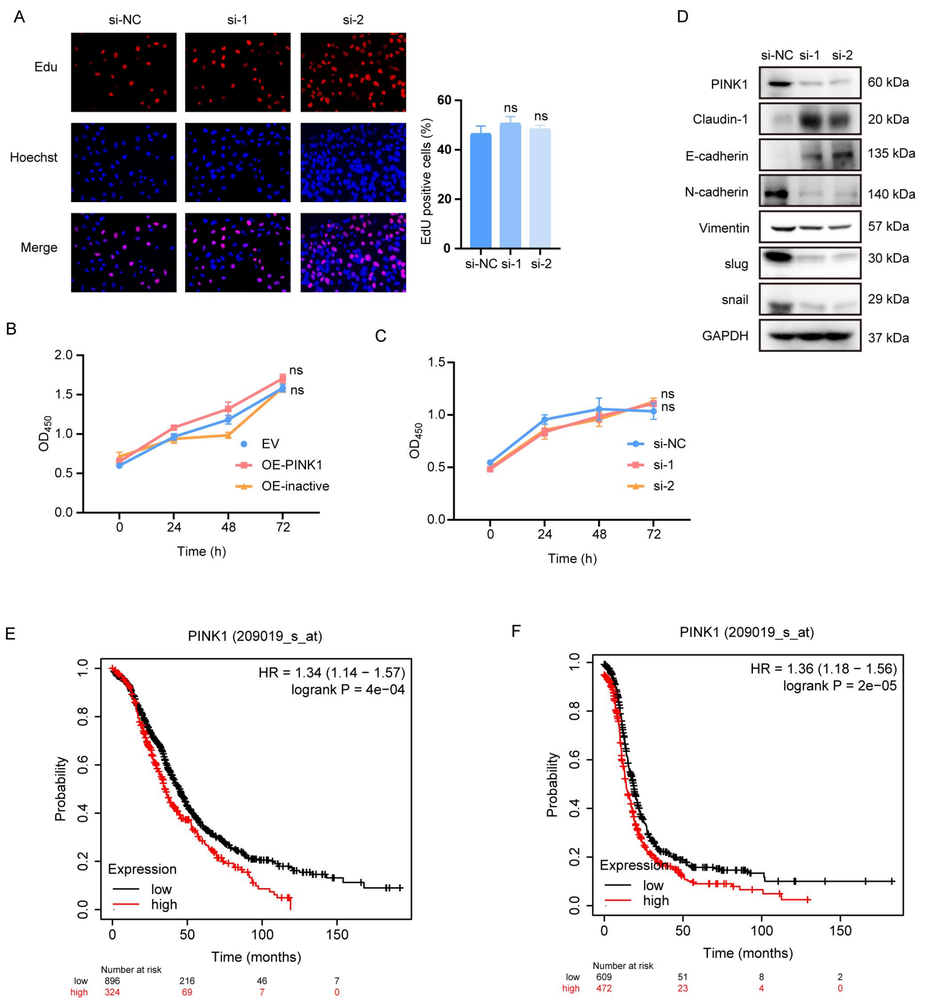

Figure S3

A Representative images (left) and quantitative analysis (right) of proliferating SKOV3 cells, which were transfected by siRNA targeting PINK1 for 48 h and then assessed by EdU kit assay. **B and C** Effects of PINK1 on proliferation ability of A2780 and SKOV3 cells. A2780 (**B**) and SKOV3 (**C**) cells were transfected by PINK1 over-expression plasmid and siRNA targeting PINK1 respectively for 24, 48, and 72 h, and then assessed by CCK-8 kit assay. **D** Western blot analysis of EMT markers expression. SKOV3 cells were transfected by PINK1-targeting siRNA. **E and F** An analysis of Overall Survival

(**E**) and Progression-Free Survival (**F**) curves in Stage 3+4 human ovarian cancer, utilizing data available on the Kaplan-Meier Plot website. All relevant data on the website were stratified according to PINK1 expression. ns, no statistical significance. Values are mean ± SEM. Data are representative of 3 independent experiments.

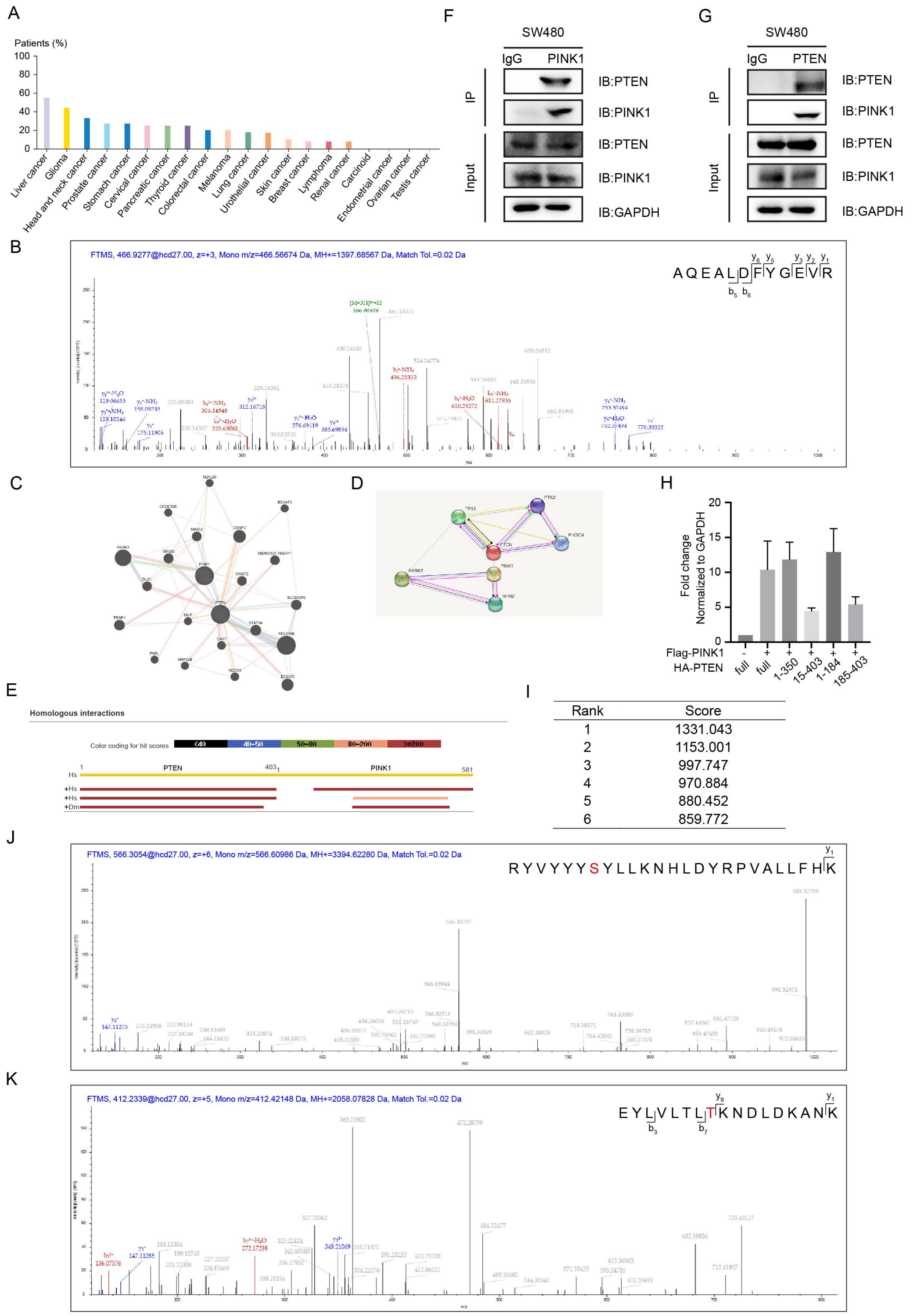

Figure S4

A The protein expression level of PRKN in diverse tissues downloaded from The Human Protein Atlas. **B** PTEN was detected by mass spectrometry

analysis. **C** Interaction prediction of PINK1 and PTEN by genemania database. **D** Interaction prediction of PINK1 and PTEN by string database. **E** Interaction prediction of PINK1 and PTEN by hitpredict analysis. **F and G** Western blot analysis of endogenous PINK1 and PTEN expression in a co-IP assay performed in SW480 cells with Protein A/G PLUS-Agarose and anti-PINK1 (**F**) or anti-PTEN (**G**) primary antibody. **H** Quantification analysis of PINK1 interacted with PTEN in Figure 3J. **I** The Z-DOCK score of PINK1 and PTEN interaction simulation models. **J and K** Phosphorylated Ser179 (J) and Thr321 (K) residue in PTEN detected by mass spectrometry analysis of SKOV3 cells.

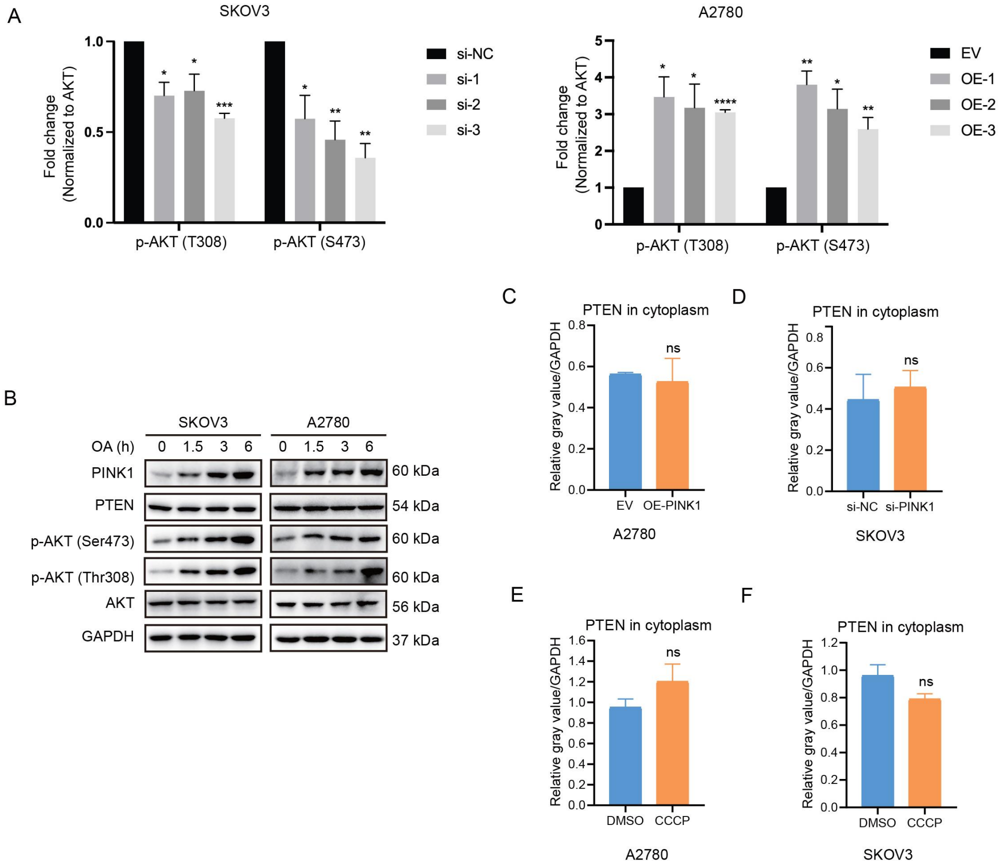

Figure S5

A Quantification of p-AKT in Figure 5A. **B** Western blot analysis of p-AKT in A2780 and SKOV3 cells treated with oligomycin and antimycin (OA), a mitophagy inducer, for indicated time. **C and D** Quantification of PTEN protein level in cytoplasm in Figure **5I** and **5J**. **E and F** Quantification of PTEN protein level in cytoplasm in Figure **5K** and **5L**.
