# Supplementary material for: PINK1-PTEN axis promotes metastasis and chemoresistance in ovarian cancer via non-canonical pathway
Source: J Exp Clin Cancer Res. 2023 Nov 9;42:295. doi: 10.1186/s13046-023-02823-w (PMC10633943; doi:10.1186/s13046-023-02823-w)
Supplement: Supplementary file 2 — Additional file 2: Table S1. The detailed antibody information. Table S2. Primers and sequences. Table S3. Detailed information of included datasets from the Kaplan-Meier Plotter. Table S4. The detailed mass spectrometry information for peptidegroup. [file 13046_2023_2823_MOESM2_ESM.pdf]

Table S1. The detailed antibody information

Antibodies	Source	Cat#
Anti-GAPDH	Cell signaling Technology	#5174
Anti-PINK1	Cell signaling Technology	#6946
Anti-PTEN	Cell signaling Technology	#9599
Anti-PTEN	proteintech	60300-1-Ig
Anti-Parkin	Cell signaling Technology	#32833
Anti-Claudin-1	Cell signaling Technology	#13255
Anti-E-cadherin	Cell signaling Technology	#14472
Anti-N-cadherin	Cell signaling Technology	#13116
Anti-Vimentin	Cell signaling Technology	#5741
Anti-slug	Cell signaling Technology	#9585
Anti-snail	Cell signaling Technology	#3879
Anti-Lamin A/C	Cell signaling Technology	#4777
Anti- Phospho-AKT Thr308	Cell signaling Technology	#13038
Anti- Phospho-AKT Ser473	Cell signaling Technology	#4060
Anti-AKT	Beyotime	AF1789
Anti- pan Phospho-Serine/Threonine Rabbit Polyclonal Antibody	Beyotime	AF5725
Anti-HA	Cell signaling Technology	#3724
Anti-Flag	Cell signaling Technology	#14793
Anti-Ubiquitin	Beyotime	AF1704
Anti-CA125	Abcam	ab110640
Anti-Ki67	Beyotime	AF1738
Anti-LDHA	Beyotime	AF0216

Table S2. Primers and sequences

**Primers for PCR and real time PCR**		
Primers	Sequences-F (5' → 3')	Sequences-R (5' → 3')
PINK1	ACCTTTGCCCCTAACACGAG	AACTGAACGTGCTGACCCAT
PTEN	ACCAGGACCAGAGGAAACCT	GCTAGCCTCTGGATTTGACG
GAPDH	AGGTCGGTGTGAACGGATTTG	GGGGTCGTTGATGGCAACA
**Sequences for gene knockdown**		
si-PINK1-1	GGAGCCAUCGCCUAUGAAATT	UUUCAUAGGCGAUGGCUCCTT
si-PINK1-2	GCAAAUGUGCUUCAUCUAATT	UUAGAUGAAGCACAUUUGCTT
si-PINK1-3	GCUAACCUGGAGUGUGAAATT	UUUCACACUCCAGGUUAGCTT
si-NC	UUCUCCGAACGUGUCACGUTT	ACGUGACACGUUCGGAGAATT
sh-PINK1	CACCGCTGGAGGAGTATCTGATAGGTTCA AGAGACCTATCAGATACTCCTCCAGC	AAAAGCTGGAGGAGTATCTGATAGGTCTCT TGAACCTATCAGATACTCCTCCAGC

Table S3 Detailed information of included datasets from the Kaplan-Meier Plotter

Dataset	Included number	Histology			Stage				Geographic location	Publication year
		Endometrioid	Serous	Others	I	II	III	IV		
GSE27651	39	/	/	/	7	1	28	3	USA	2011
GSE26712	185	/	/	/	/	/	/	/	USA	2011
GSE15622	35	0	31	4	0	0	26	9	Switzerland	2009
GSE30161	58	1	47	10	0	0	53	5	USA	2012
GSE19829	28	/	/	/	/	/	/	/	USA	2010
GSE63885	75	1	70	4	0	2	63	10	Poland	2014
GSE3149	115	/	/	/	0	1	95	19	USA	2005
GSE18520	53	0	53	0	/	/	/	/	USA	2009
TCGA	557	0	557	0	15	27	427	85	USA	/
GSE14764	80	7	68	5	8	1	69	2	Germany	2009
GSE26193	107	8	79	20	20	11	59	17	France	2011
GSE9891	285	20	264	1	24	18	217	22	Australia	2008

Table S4. The detailed mass spectrometry information for peptidegroup

Sequence	Positions in Master Proteins	Modifications in Master Proteins	Theo. MH+ [Da]	Abundances	Confidence	m/z [Da]	DeltaM [ppm]
AGKGRTGVMICAYLLHRGK	sp [126-144]		2104.12664		High	351.52991	7.8
RYVYYYSYLLKNHLDYRPVALLFHK	sp [173-197]	sp 2xPhospho [Y174; S179]	3394.65778	88306.92969	High	566.60986	-10.31
RYQEDGFDLDLTYIYPNIIAMGFPAERLEGVYRNNIDDVVR	sp [15-55]	sp 1xPhospho [Y46]	4930.35461	38106.51953	High	986.8645	-12.41
RYQEDGFDLDLTYIYPNIIAMGFPAERLEGVYR	sp [15-47]	sp 1xPhospho [Y46]	4020.88778	106410.75	High	804.98291	-0.58
NNIDDVVR	sp [48-55]		944.47958	5183542	High	472.74438	2.02
NHLDYRPVALLFHKMMFETIPMFSGGTCNPQFVVCQLK	sp [184-221]		4558.20009		High	760.53979	0.5
MTAIIK	sp [1-6]		676.40621	351535.7031	High	676.40698	1.14
IYSSNSGPTR	sp [224-233]		1081.52726	6495780.5	High	541.26837	2.04
VEFFHK	sp [255-260]		806.41955	2258414.672	High	403.71445	2.56
LEGVYRNNIDDVVRFLDSK	sp [42-60]		2252.16697		High	451.24548	13.92
KGVTIPSQR	sp [164-172]		985.5789	309714.5313	High	493.29486	3.59
HYDTAK	sp [75-80]		734.34678		High	734.34204	-6.45
GRTGVMICAYLLHRGKFLK	sp [129-147]		2236.22054	263041	High	448.04703	-6.49
GRTGVMICAYLLHRGK	sp [129-144]		1847.9731	818893.25	High	616.6615	-1.71
FLDSKHKNHYKIYNLCAER	sp [56-74]		2436.22411		High	406.8728	-9.72
EYLVLTLTKNDLDKANK	sp [314-330]	sp 1xPhospho [T321]	2058.05186		High	412.42148	12.84
AQEALDFYGEVR	sp [148-159]		1397.66957	780159.75	High	699.34192	5
LEGVYR	sp [42-47]		736.39882	8136344.688	High	368.7037	1.79
YFSPNFK	sp [336-342]		902.44068	4122346.25	High	451.72641	5.39
