# Supplementary material for: PINK1-PTEN axis promotes metastasis and chemoresistance in ovarian cancer via non-canonical pathway
Source: J Exp Clin Cancer Res. 2023 Nov 9;42:295. doi: 10.1186/s13046-023-02823-w (PMC10633943; doi:10.1186/s13046-023-02823-w)
Supplement: Supplementary file 3 — Additional file 3. Supplementary material for methods. [file 13046_2023_2823_MOESM3_ESM.pdf]

Supplementary material for Methods

1. Cell lines and cell culture

Human ovarian cancer A2780 cells were purchased from iCell Bioscience Inc (Shanghai, China). Human ovarian cancer cell lines SKOV3, ES-2, OVCAR3 and human epithelial kidney cell HEK293T were obtained from the Culture Collection of the Chinese Academy of Sciences (Shanghai, China). Normal ovarian epithelial cell line IOSE80 was purchased from the BeNa Culture Collection (Hebei, China). SH-SY5Y cells were obtained from the American Type Culture Collection (ATCC). These cells were authenticated by DNA (STR) profiling by iCell Bioscience Inc (Shanghai, China). Human cisplatin-resistant cell line SKOV3/DDP was developed from SKOV3 by chronic exposure to cisplatin. SKOV3 and SKOV3/DDP cells were maintained in McCoy's 5A medium supplemented with 10% fetal bovine serum. A2780 cells, ES-2 cells, SH-SY5Y cells and HEK293T cells were cultured with Dulbecco's modified Eagle's medium (DMEM) containing 10% fetal bovine serum. OVCAR3 cells were cultured with RPMI 1640 medium with 20% fetal bovine serum and 0.01mg/ml bovine insulin. All medium were supplemented with 1% penicillin-streptomycin and incubated in 37°C, 5% CO_2 condition.

2. Cell proliferation assay

The proliferation ability of ovarian cancer cells was assessed by the EdU (5-ethynyl-2'-deoxyuridine) Cell Proliferation Kit (C0078, Beyotime, China) and Cell counting Kit-8 (CCK-8) assay (C0037, Beyotime, China). In CCK-8 assay,

2 × 10^4 cells were seeded in each well of the 96-well plate and cultured for 24, 48, and 72 h. After incubating with CCK-8 solution at 37°C for 1 h, the absorbance at 450 nm of each well was measured to reflect the corresponding proliferation capacity of cells. Experiments were conducted in triplicate.

For EdU assay, pre-treated SKOV3 and A2780 cells were seeded in 6-well plates at a density of 2.5 × 10^5/ well. After 48 h, cells were added EdU reagent for 2 h at 37°C and then fixed with 4% paraformaldehyde, followed by Hoechst 33 342 staining for nuclear for 10 min. The image acquisition was applied with a fluorescence microscopy (Nikon). The percentage of EdU-positive cells was regarded as the proliferation rate.

3. Cell Transwell assay and Wound healing assay

The Transwell assay with 8-μm Transwell Supports (Corning, USA) was used to evaluate the cell invasion and migration ability. Pre-treated A2780 or SKOV3 cells in 100μl serum-free medium were seeded into the upper chamber pre-coated with (for invasion assay) or without (for migration assay) Matrigel Matrix (356234, Corning). After incubation of 24 h with 600μl medium containing 10% serum in the lower chamber, cells that did not invade or migrate were removed by a cotton swab. Then the invading or migrating cells were fixed with 4% paraformaldehyde on the bottom surface of the membrane for 10 min. Next, 0.4% crystal violet solution was used to stain cells. Cells were

thoroughly washed with PBS and photographed with a digital microscopy (Carl Zeiss Jena, Germany). The number of invading or migrating cells was counted in 6 randomly selected fields for statistical analysis. Experiments were performed in triplicate.

For wound scratch assay, pre-treated A2780 or SKOV3 cells were seeded in 6-well plate. While the cells reached 100% confluence, monolayer cells were wounded. Then the wells were rinsed three times and serum-free medium was used instead.

4. Public datasets retrieval

The datasets used in Kaplan-Meier Plotter is shown as following: TCGA, GSE14764, GSE 15622, GSE18520, GSE19829, GSE23554, GSE26193, GSE26712, GSE27651, GSE30161, GSE3149, GSE51373, GSE63885, GSE65986, GSE9891.

5. Western blotting

Protein lysates were separated on 8%-15% SDS-PAGE gels and then transferred to nitrocellulose filter membranes. Next, membranes were blocking with No protein blocking solution, 2% (C530040-0500, Sangon Biotech) in Tris-buffered saline and incubated overnight with primary antibodies in 4 °C. The next day, the membranes were incubated with secondary horseradish peroxidase-conjugated anti-rabbit or anti-mouse IgG antibodies for 2 h and the specific bands were visualized by ChemiDoc^{TM} Touch Imaging System

(Bio-Rag, USA) with the ECL substrate (P0018FS, Beyotime). Grayscale values of corresponding bands were measured by ImageJ software to quantify the expression of proteins. GAPDH served as a loading control while Lamin A/C was used as loading control for nuclear protein. Experiments were performed in triplicate.

6. Quantitative real-time PCR

Trizol reagent (B511311, Sangon Biotech, China) was applied to extract total RNA from cells. After extraction according to manufacturer's instructions, RNA was reverse transcribed to cDNA using PrimeScript™ RT reagent Kit with gDNA Eraser (RR047A, Takara, Japan). Then cDNA primers specific for different mRNAs were used in quantitative real-time PCR. The gene GAPDH served as an internal control. Primer sequence was shown in **Supplementary material, Table S2**. All these quantitative real-time PCR reactions were performed with Takara's SYBR Premix Ex Taq™ II (Tli RNaseH Plus) in Applied Biosystems 7500 Fast Real-Time PCR System (Applied Biosystems, USA). The $2^{-\triangle\triangle Ct}$ method was applied to quantification and the gene GAPDH was adopted to calculate the fold change. Experiments were performed in triplicate.

7. Immunofluorescence staining

Pre-treated A2780, SKOV3 or HEK293T cells were seeded into 6-well plate

and grown on sterilized coverslips. After the cells were attached the coverslips, cells were fixed by 4% paraformaldehyde for 30 min, then permeabilized with 1% Triton X-100 (Sangon Biotech, China) for 10 min. Next, cells were blocked by No protein blocking solution, 2% (C530040-0500, Sangon Biotech) for 1 h and incubated with primary antibodies at 4°C overnight, including PINK1 (#6946, 1:200, Cell Signaling Technology), PTEN (60300-1-Ig, 1:500, proteintech). The next day, cells were incubated with fluorochrome-labeled anti-rabbit secondary antibody (1:100, MultiSciences) for 2 h. The coverslips were then stained with DAPI (1:10 000, Beyotime) and the image acquisition was applied with a fluorescence microscopy (bx63, Olympus, Japan) or Inverted laser scanning Confocal microscope (FV1000, Olympus, Japan).

8. *In vitro* kinase assay

In vitro kinase assay were performed with 20μl of affinity-purified PINK1 and a 10μl volume of affinity-purified substrate (PTEN) in a reaction volume of 50μl, consisting of 20μl kinase buffer (#9802, Cell Signaling Technology) with or without 200μM ATP. After a reaction at 30°C for 30 min, sample buffer was added for the termination. Then the samples were boiled for 10 min and analyzed via SDS-PAGE.

9. Cytoplasmic and nuclear protein extraction

The cells were collected and lysed with NP-40 Lysis Buffer as described above.

Cytoplasmic and nuclear protein of cells was separated by Nuclear and Cytoplasmic Protein Extraction Kit (P0027, Beyotime) according to the manufacturer's instruction.

10. Flow cytometry analysis

SKOV3/DDP cells were treated with different concentrations of DDP for 48 h, followed by harvest and washed twice with PBS. Then the cells were fixed with 70% ethanol at 4°C overnight. Annexin V-FITC/PI apoptosis kit (AP101-100, MultiSciences, China) was used to detect the apoptotic cells according to the manufacturer’s protocol. Flow cytometry analysis was conducted using a Dxflex flow cytometer.

11. Co- Immunoprecipitation (Co-IP) assay

Cells were lysed with NP-40 Lysis Buffer (P0013F, Beyotime, China, containing 50mM Tris pH7.4, 150mM NaCl, 1% NP-40, sodium pyrophosphate, β- glycerophosphate, sodium orthovanadate, sodium fluoride, EDTA, leupeptinfor) with complete protease and phosphatase inhibitors for 30 min. Then the lysates were precleaned with Protein A/G PLUS-Agarose (sc-2003, Santa Cruz) or corresponding IgG for 2 h at 4 °C. Pre-cleaned lysates were immunoprecipitated with the indicated antibodies overnight at 4 °C. The next day, the lysates were incubated with Protein A/G PLUS-Agarose for 2 h at 4 °C. After washing four times with washing buffer of beads, the bound proteins

were eluted by boiling at 100 °C on block heater for 15 min, followed by Western blot.

12. Construction and transfection of plasmid, siRNA, and short hairpin RNA

The small interfering RNA (siRNA) was transfected using Powerfect reagent (SL100569, SignaGen, USA) according to the manufacturer's protocol. The siRNAs targeting PINK1 (si-PINK1) and negative control (si-NC) were obtained from GenePharma (China). A2780, SKOV3, and HEK293T cells were transfected for 48 h with si-PINK1. The sequences of siRNAs were shown in **Supplementary material, Table S2**. The full-length and PTEN-truncated plasmids were transfected using Lipofectamine 3000 (Thermo Fisher Scientific, USA).

Short hairpin RNA (shRNA) was used to construct stable PINK1 knockdown in SKOV3 cells, and the sequence of shRNAs were listed in **Supplementary material, Table S2.** HEK293T cells were used to transfect plasmid (plvx-shRNA2) and packaging vectors (pMD2.G and psPAX2) by Lipo3000. The medium supernatant containing lentivirus particles was collected after 48 h and filtered through 0.45μm filter. And the virus was added to infect SKOV3 cells together with 7μg/ml Polybrene (C0351, Beyotime, China). Then these cells were selected after 48 h by 1μg/mL puromycin (ST551, Beyotime, China)

for 5 days to obtain cells with PINK1 stably silenced.

Site mutant plasmids were constructed by overlap PCR and the primers were designed by Takara Primer design tool (https://www.takarabio.com/learning-centers/cloning/primer-design-and-other-tools).

13. Mass spectrometry analysis and Protein structure analysis

To identify the interacting protein of PINK1, the total proteins were immunoprecipitated from SKOV3 cells with anti-PINK1 primary antibody and Protein A/G PLUS-Agarose (sc-2003, Santa Cruz). Immunoprecipitates were separated by SDS-PAGE and stained with Coomassie brilliant blue. The bands were excised and the protein were precipitated and digested, followed by analysis by liquid chromatograpgy-tandem mass spectrometry (Beijing Qinglian Biotech Co.,Ltd, China).

To identify phosphorylation sites of PTEN by PINK1, Flag-PINK1 was over-expressed in SKOV3 cells and SKOV3 lysates were immunoprecipitated with anti-Flag antibody. Immunoprecipitates were separated by SDS-PAGE and stained with Coomassie brilliant blue. The corresponding size of bands were excised and subjected to mass spectrometry (Beijing Qinglian Biotech Co.,Ltd, China). Phosphopeptides were analyzed by the Q Exactive LC-MS/MS system (Thermo Scientific). All identified phosphorylation sites were checked manually.

Z-docking server was used for protein structure analysis and simulating molecular docking. The results were visualized in Pymol (The PyMOL Molecular Graphics System, Version 2.0 Schrödinger, LLC).

14. Immunohistochemistry (IHC) staining

Paraffin-embedded samples were performed IHC using SP Rabbit & Mouse HRP Kit (DAB) (CW2069S, CWBIO, China). After deparaffinization in dimethylbenzene and a graded ethanol series and antigen retrieval, samples were blocked in blocking solution for 1 h. Then the sections were incubated with the primary antibody at 4°C overnight, followed by Goat anti-Rabbit/Mouse IgG, Biotin and Streptavidin-HRP at room temperature. Next the chromogenic reaction was conducted using DAB. Then the sections were counterstained with hematoxylin for 5 min and the image acquisition was applied with a Bx63 Olympus microscope (Olympus, Japan). Then immunoreactivity was scored based on the percentage of positively stained ovarian cells and the intensity of staining by two investigates respectively.
